# A Robust ConvNeXt-Based Framework for Efficient, Generalizable, and Explainable Brain Tumor Classification on MRI

**DOI:** 10.3390/bioengineering13020157

**Published:** 2026-01-28

**Authors:** Kirti Pant, Pijush Kanti Dutta Pramanik, Zhongming Zhao

**Affiliations:** 1 Department of Computer Science and Engineering, Bipin Tripathi Kumaon Institute of Technology, Dwarahat 263653, Uttarakhand, India; pantkirti577@gmail.com; 2 School of Computer Applications and Technology, Galgotias University, Greater Noida 203201, Uttar Pradesh, India; 3 Center for Precision Health, McWilliams School of Biomedical Informatics, The University of Texas Health Science Center at Houston, Houston, TX 77030, USA

**Keywords:** brain tumor classification, magnetic resonance imaging, ConvNeXt, deep learning, explainable AI, medical image analysis, cross-dataset generalization, statistical validation

## Abstract

**Background:** Accurate and dependable brain tumor classification from magnetic resonance imaging (MRI) is essential for clinical decision support, yet remains challenging due to inter-dataset variability, heterogeneous tumor appearances, and limited generalization of many deep learning models. Existing studies often rely on single-dataset evaluation, insufficient statistical validation, or lack interpretability, which restricts their clinical reliability and real-world deployment. **Methods:** This study proposes a robust brain tumor classification framework based on the ConvNeXt Base architecture. The model is evaluated across three independent MRI datasets comprising four classes—glioma, meningioma, pituitary tumor, and no tumor. Performance is assessed using class-wise and aggregate metrics, including accuracy, precision, recall, F1-score, AUC, and Cohen’s Kappa. The experimental analysis is complemented by ablation studies, computational efficiency evaluation, and rigorous statistical validation using Friedman’s aligned ranks test, Holm and Wilcoxon post hoc tests, Kendall’s W, critical difference diagrams, and TOPSIS-based multi-criteria ranking. Model interpretability is examined using Grad-CAM++ and Gradient SHAP. **Results:** ConvNeXt Base consistently achieves near-perfect classification performance across all datasets, with accuracies exceeding 99.6% and AUC values approaching 1.0, while maintaining balanced class-wise behavior. Statistical analyses confirm that the observed performance gains over competing architectures are significant and reproducible. Efficiency results demonstrate favorable inference speed and resource usage, and explainability analyses show that predictions are driven by tumor-relevant regions. **Conclusions:** The results demonstrate that ConvNeXt Base provides a reliable, generalizable, and explainable solution for MRI-based brain tumor classification. Its strong diagnostic accuracy, statistical robustness, and computational efficiency support its suitability for integration into real-world clinical and diagnostic workflows.

## 1. Introduction

**Figure 1:**
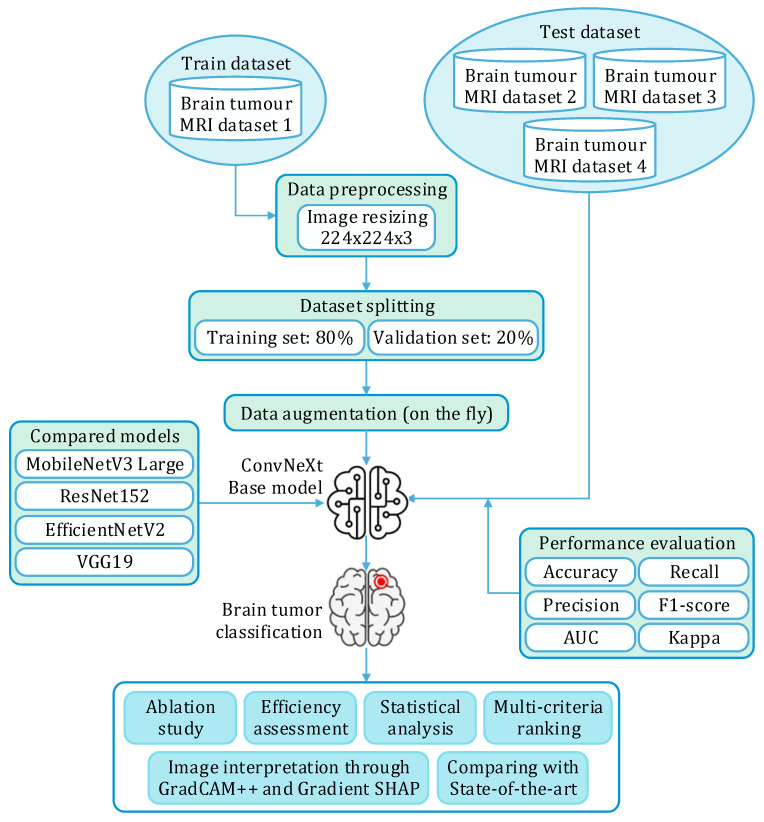
Research methodology.

Brain tumors represent one of the most severe and life-threatening forms of cancer due to their aggressive growth, complex pathology, and disruption of critical neurological functions. Globally, they rank among the top ten causes of cancer-related mortality in both adults and children, contributing substantially to the overall disease burden. According to the World Health Organization (WHO), marked regional disparities exist in incidence and mortality rates, with Europe and North America experiencing the highest burden, while Asia and Africa report comparatively lower figures [1]. Nevertheless, across all regions, the persistent gap between incidence and mortality highlights the limited survival gains achieved even in well-resourced healthcare systems. Importantly, brain tumors disproportionately affect younger populations and are associated with one of the highest standardized mortality ratios (SMRs) among all cancers, often persisting decades after diagnosis [2]. These trends underscore the urgent need for improved diagnostic strategies, earlier intervention, and more equitable neuro-oncology care worldwide.

Brain tumors arise from abnormal and uncontrolled proliferation of cells within the brain and its surrounding structures, including the meninges, cranial nerves, and pituitary gland. They are broadly classified into primary tumors, originating within the brain, and secondary or metastatic tumors that spread from other organs. Among primary tumors, gliomas, meningiomas, and pituitary tumors are the most prevalent, with gliomas exhibiting particularly invasive and aggressive behaviour [3]. Risk factors associated with brain tumor development include genetic predisposition, exposure to ionizing radiation, carcinogenic chemicals, viral infections, and inherited syndromes such as Li–Fraumeni and neurofibromatosis. Clinically, patients often present with non-specific neurological symptoms—such as headaches, seizures, visual disturbances, or cognitive impairment—which complicate early and accurate diagnosis [3]. As a result, reliable and efficient diagnostic tools are critical for improving clinical outcomes.

Medical imaging plays a central role in the diagnosis and management of brain tumors. Among available modalities, magnetic resonance imaging (MRI) offers superior soft-tissue contrast and detailed visualization of intracranial structures, making it the gold standard for tumor detection, localization, and treatment planning [4]. Despite its diagnostic value, manual interpretation of MRI scans remains time-intensive and subject to inter-observer variability. Subtle tumor boundaries, infiltrative growth patterns, and class-specific visual similarities may not always be readily apparent, potentially delaying treatment decisions. To mitigate these challenges, artificial intelligence (AI)-driven approaches—particularly deep learning—have gained increasing attention for automated tumor detection, classification, and segmentation. By learning discriminative features directly from imaging data, deep learning models have demonstrated notable improvements in diagnostic accuracy, reproducibility, and efficiency [5].

Among AI techniques, convolutional neural networks (CNNs) have emerged as the backbone of modern medical image analysis due to their ability to learn hierarchical spatial representations [6,7]. CNN-based models have been successfully applied to brain tumor classification, grading, and segmentation, often outperforming traditional machine-learning approaches reliant on hand-crafted features [8]. The adoption of transfer learning, in which CNNs pretrained on large-scale natural image datasets are fine-tuned on medical images, has further accelerated progress by reducing data requirements and training time while improving generalization [9]. However, conventional CNN architectures remain limited by their inherently local receptive fields, which restrict their ability to capture long-range contextual relationships—an important factor when distinguishing tumors with heterogeneous morphology or diffuse boundaries [10].

To address these limitations, recent studies have explored architectures inspired by Vision Transformers (ViTs), which leverage self-attention mechanisms to model global dependencies [11,12]. While ViTs have demonstrated promising results in medical imaging, they typically require large datasets and substantial computational resources, posing practical challenges for routine clinical use. This has motivated interest in hybrid and next-generation convolutional architectures that retain the efficiency of CNNs while incorporating design principles inspired by transformers.

Despite substantial progress in various deep learning family-based brain tumor classification, several gaps remain in the current literature. Many studies report strong performance on a single dataset but do not assess robustness under cross-dataset evaluation, limiting confidence in real-world generalization. Statistical validation of performance differences is often omitted, making it unclear whether reported gains are meaningful or incidental. Furthermore, computational efficiency and deployment feasibility are rarely analyzed, even though these factors strongly influence clinical adoption. Finally, interpretability is frequently addressed in isolation or not at all, leaving uncertainty about whether model decisions align with radiologically meaningful regions.

Within this context, the present study investigates ConvNeXt Base as a modernized convolutional architecture that combines the representational strength of transformer-inspired designs with the efficiency of classical CNNs. ConvNeXt modernizes the ResNet design by integrating large-kernel depthwise convolutions, inverted bottlenecks, and layer normalization. These design choices enable ConvNeXt to capture both local and global image features while preserving computational efficiency.

While ConvNeXt has shown strong performance in natural image benchmarks, its potential for brain tumor MRI classification remains underexplored. This work addresses that gap by investigating the effectiveness of the ConvNeXt Base architecture for multi-class brain tumor classification using MRI images across four tumor categories. The choice of ConvNeXt is motivated by its ability to combine transformer-inspired global contextual modeling with the robustness and efficiency of convolutional operations, making it well suited for complex medical imaging tasks. We rigorously evaluate its performance across multiple independent datasets, examine its generalization capability, and assess interpretability using post-hoc explainability techniques.

The scope of this work is focused on the reliability and robustness of brain tumor classification systems rather than on tumor segmentation, grading, or molecular characterization. While ConvNeXt has emerged as a modernized convolutional architecture that blends CNN efficiency with transformer-inspired scalability, its potential in medical imaging remains relatively underexplored [13]. The novelty of this study lies not in proposing a new architecture, but in delivering a comprehensive and evidence-driven evaluation of ConvNeXt Base under clinically relevant conditions.

Specifically, we address the critical issue of external generalization by training and validating the model across three independent datasets, a protocol that remains uncommon in prior studies [14,15]. Furthermore, ConvNeXt Base is benchmarked against a diverse set of established transfer-learning architectures, including lightweight, scalable, residual, and classical deep CNNs, enabling a fair and holistic comparison that many existing works do not emphasize [16]. In addition, extensive ablation experiments are conducted to disentangle the contributions of data augmentation strategies, optimizer choices, and architectural parameters, thereby offering practical design insights for future medical imaging research [17]. Interpretability is enhanced through a dual explainability framework that integrates Grad-CAM++ and Gradient SHAP, a combination rarely explored together in brain tumor analysis. Finally, reproducibility and deployment feasibility are reinforced through rigorous statistical validation, resource-efficient augmentation, and efficiency profiling.

Although three-dimensional (3D) and multimodal MRI analyses can capture richer spatial and contextual information, this study adopts a two-dimensional (2D) slice-based formulation by design. This choice reflects practical constraints commonly encountered in clinical and public datasets, including heterogeneous acquisition protocols, inconsistent volumetric coverage, and limited availability of fully annotated 3D data. A 2D formulation enables uniform preprocessing across datasets and supports computationally efficient training and inference, which is essential for scalable clinical deployment. Moreover, prior studies have demonstrated that well-optimized 2D deep learning models can achieve diagnostic performance comparable to volumetric approaches for tumor classification tasks. Extensions to 3D and multimodal MRI inputs are therefore positioned as a logical continuation of this work rather than a prerequisite for its core objectives.

The main contributions of this study are summarized as follows:

- Adaptation and optimization of ConvNeXt Base for medical imaging, demonstrating strong generalization without architectural over-complexity.
- Comprehensive cross-dataset validation across three independent MRI datasets to assess robustness beyond single-domain evaluation.
- Extensive benchmarking against modern and classical CNN architectures under identical experimental settings.
- Detailed ablation studies examining the effects of transfer learning, attention mechanisms, data augmentation, and input resolution.
- Rigorous statistical significance testing using Friedman, Holm, Wilcoxon, and Kendall’s W analyses with critical difference diagrams.
- Computational efficiency profiling, including inference time, resource utilization, and power consumption.
- Multi-perspective interpretability analysis using Grad-CAM++ and Gradient SHAP to confirm tumor-relevant decision-making.
- Clinically oriented evaluation using sensitivity, specificity, precision, recall, F1-score, AUC, Kappa metrics, and confusion-matrix visualization.

The remainder of this paper is organized as follows: Section 2 reviews related work in deep learning-based brain tumor classification. Section 3 describes the ConvNeXt Base architecture, experimental setup, datasets, preprocessing pipeline, and evaluation metrics. Section 4 presents the results, including performance analysis, ablation studies, explainability, efficiency assessment, and statistical validation. Section 5 discusses the findings and their clinical implications. Section 6 outlines study limitations and future research directions, and Section 7 concludes this paper.

## 2. Related Work

Prior studies on MRI-based brain tumor classification have explored a wide range of deep learning strategies, differing substantially in architectural design, evaluation rigor, and validation scope. Early works primarily relied on classical convolutional backbones trained via transfer learning, while more recent studies have introduced attention mechanisms, transformer variants, and hybrid CNN–transformer architectures. Despite reporting high classification accuracies, many of these studies are limited by single-dataset evaluation, incomplete metric reporting, or a lack of statistical validation and interpretability analysis. This section critically reviews representative approaches in the literature, focusing on how architectural choices, training strategies, validation protocols, and explainability practices influence reported performance and real-world applicability.

### 2.1. Traditional CNN-Based Approaches

CNNs have long been the cornerstone of automated brain tumor analysis, demonstrating high sensitivity and specificity in diagnostic tasks. For instance, Raza et al. [18] developed an automated CNN framework for early detection that integrated advanced preprocessing and achieved superior classification over conventional approaches. Similarly, Ahamed and Sadia [19] proposed multimodal MRI augmentation using residual learning, effectively addressing vanishing gradients and enhancing robustness. Stathopoulos et al. [20] benchmarked multiple MRI modalities and CNN backbones on 1646 slices, achieving up to 98.6% accuracy, while also providing modality-specific insights for clinical screening. These works underline CNNs’ role as foundational yet still limited in their capacity to capture long-range dependencies.

### 2.2. Transfer Learning and Pre-Trained CNNs

Given the persistent challenges of limited labeled data and heterogeneity across MRI acquisitions, transfer learning—fine-tuning ImageNet-pretrained backbones—has become the de facto strategy for brain tumor classification and related tasks. Several comparative studies confirm the utility of this approach: Aggarwal et al. [21] empirically showed that fine-tuning established backbones such as VGG16, ResNet50, and InceptionV3 yields consistent gains in both accuracy and training efficiency, while Pravitasari et al. [22] directly contrasted pre-training with random initialization and identified EfficientNet-B5 (with pretrained weights) as particularly effective for both binary and multi-class tumor problems. Building on these baseline findings, many works explored both architectural choices and hybridizations to squeeze extra performance: Nayak et al. [23] fused DenseNet and EfficientNet into a hybrid pipeline to capture complementary representations, and Mathivanan et al. [9] performed a head-to-head evaluation of ResNet152, VGG19, DenseNet169, and MobileNetV3 on a balanced corpus, reporting that MobileNetV3 offered the best accuracy–efficiency tradeoff in their setting. Other studies targeted optimizer and training refinements—Polat & Güngen [24] found ResNet50 with the Adadelta optimizer effective on the Figshare MRI benchmark, and Mehnatkesh et al. [25] leveraged metaheuristic search to tune transfer-learning pipelines for improved generalization. Practical concerns of data scarcity and robustness also motivated non-standard pretraining and augmentation strategies: Ghassemi et al. [26] used a GAN discriminator as a pre-trained classifier and combined heavy augmentation with dropout to better resist overfitting, while Salama & Shokry [27] (and related synthetic-augmentation studies) reported that synthetic samples can help rebalance small or skewed MRI collections. Several groups focused on architectural tweaks and attention mechanisms to localize tumor cues more effectively: Shaik & Cherukuri [28] proposed MANet, a multi-level attention model built on Xception that concentrates on salient tumor regions, Sharif et al. [29] applied DenseNet201 to classify four tumor types, and Sharma et al. [30] enhanced ResNet50 with extra layers and careful fine-tuning to boost multi-class discrimination. Complementary empirical studies—Alanazi et al. [31] on cross-device subclassification and Vimala et al. [32] fine-tuning EfficientNet variants with Grad-CAM visualizations—underscore that transfer learning is not only effective but can be made more interpretable and device-robust with suitable training and visualization choices. Additional studies have extended transfer learning pipelines with large-scale datasets and multi-class frameworks: Zarina et al. [33] applied a hierarchical multiscale deformable attention module (MS-DAM) to 14 tumor types, achieving >96.5% accuracy, while Wong et al. [34] leveraged pretrained VGG16 with augmented datasets of 17,136 MRI images, reporting 99.24% accuracy and deploying a web-based diagnostic interface. Rasa et al. [35] compared six pretrained CNNs (VGG16, ResNet50, MobileNetV2, DenseNet201, EfficientNetB3, and InceptionV3) for both binary and multi-class tasks, achieving up to 99.96% accuracy with cross-validation and demonstrating the efficiency of optimized preprocessing and augmentation strategies. Finally, large-scale comparative and optimization efforts such as those by Prabha et al. [36], Korani et al. [37], and Kumar et al. [38] demonstrate that thoughtful pooling strategies, hybrid backbones, and streamlined networks (Inception/Xception variants) often close the performance gap to heavier architectures while reducing computation.

### 2.3. Transformers for Brain Tumor Classification

The application of transformers to brain tumor analysis represents a methodological shift from purely convolutional designs toward architectures capable of capturing long-range dependencies and global context. Early CNN-based approaches often struggled to model the complex heterogeneity of tumor regions, but transformer modules have mitigated these limitations through self-attention mechanisms.

Wang et al. [39] laid the foundation with TransBTS, which embedded a transformer within a 3D CNN framework. By converting volumetric CNN features into tokens, the network achieved global context modeling while maintaining local spatial fidelity, yielding performance on par with or better than state-of-the-art methods on BraTS 2019–2020. Building on this hybridization strategy, Jiang et al. [40] introduced SwinBTS, which employs a 3D Swin Transformer as both encoder and decoder within an encoder–decoder pipeline. This hierarchical design enables effective contextual extraction while convolutional blocks refine local details, leading to consistent improvements across BraTS 2019–2021 benchmarks. In parallel, Hatamizadeh et al. [10] presented Swin UNETR, reframing segmentation as a sequence-to-sequence prediction problem. By leveraging shifted-window attention in a hierarchical encoder and connecting features to a CNN-based decoder via skip connections, Swin UNETR achieved top performance in the BraTS 2021 challenge.

While hybrid models integrate transformers with CNNs, Vision Transformers (ViTs) attempt a more radical departure by treating medical images as sequences of patches, enabling end-to-end modeling of local–global interactions. Their patch-wise representation, combined with self-attention, has proven highly effective for both classification and segmentation.

Several studies highlight the robustness of ViTs for classification tasks. Tummala et al. [41] fine-tuned multiple ViT variants (B/16, B/32, L/16, L/32) on the Figshare dataset, reporting that model ensembles significantly improved performance, achieving 98.7% accuracy. Asiri et al. [42] conducted a comparative evaluation of five pre-trained ViTs (R50-ViT-l16, ViT-l16, ViT-l32, ViT-b16, ViT-b32), confirming their adaptability across configurations, while Asiri et al. [43] introduced FT-ViT, a refined framework that integrates patch processing and multi-stage fine-tuning, improving detection accuracy in CE-MRI scans. Similarly, Reddy et al. [44] benchmarked fine-tuned ViTs against CNN backbones such as ResNet-50 and EfficientNet-B0, showing that ViTs consistently surpassed CNNs in classification accuracy.

Efforts have also extended ViTs to segmentation. Qiu et al. [45] proposed MMMViT, a multiscale multimodal design that decouples intra- and inter-modal fusion, enhancing adaptability to tumors of varying sizes and achieving strong results on BraTS 2018. Zeng et al. [46] advanced this further with DBTrans, a dual-branch architecture combining shifted-window self-attention and shuffle cross-attention with channel-attention refinements, enabling robust feature capture in both encoder and decoder stages. More recently, Poornam and Angelina [47] introduced VITALT, a composite framework integrating ViT with a Split Bidirectional Feature Pyramid Network, linear transformation, and soft quantization modules, demonstrating enhanced feature representation and robustness.

### 2.4. ConvNeXT for Brain Tumor Classification

Recent work has highlighted the promise of ConvNeXT, a modern CNN architecture inspired by ViTs, for brain tumor classification and segmentation. These studies emphasize its ability to achieve state-of-the-art performance while addressing challenges such as data scarcity, feature representation, and computational efficiency.

Bhatti et al. [48] and Mehmood & Bajwa [13] independently proposed ConvNeXT-based pipelines for brain tumor grade classification using MRI data from the BraTS 2019 dataset. Both approaches extract discriminative features from pre-trained ConvNeXT models and employ fully connected neural networks for final classification. By leveraging transfer learning, they successfully mitigated overfitting issues common in medical imaging with limited data. Their models achieved a remarkable accuracy of 99.5%, particularly when multi-sequence MRI inputs were fused as three-channel images, demonstrating ConvNeXT’s potential for robust and non-invasive tumor grading.

Extending ConvNeXT with attention mechanisms, Fırat & Üzen [49] introduced a hybrid model that integrates a spatial attention mechanism (SAM) with ConvNeXT to classify glioma, meningioma, and pituitary tumors. The combination of ConvNeXT’s enhanced receptive field and SAM’s region-focused learning improved the model’s discriminative ability, yielding 99.39% and 98.86% accuracy on the BSF and Figshare datasets, respectively. These results underscore the advantages of selectively emphasizing informative regions when classifying complex tumor structures.

Further hybridization strategies have been explored to exploit complementary architectures. Panthakkan et al. [50] proposed a concatenated EfficientNet–ConvNeXT model, demonstrating superior accuracy, sensitivity, and specificity compared to standalone models. Their framework achieved 99% predictive accuracy across multiple tumor types, while also showing robustness to variations in tumor morphology and image quality—an essential factor for clinical applicability.

Other studies have compared ConvNeXT against a broad spectrum of deep architectures. Reyes & Sánchez [16] evaluated ConvNeXT alongside VGG, ResNet, MobileNet, and EfficientNet on two MRI datasets encompassing over 3000 images. Although ConvNeXT achieved competitive accuracy (up to 98.7%), it was slower to train and had the lowest image throughput (97.35 images/s), reflecting a trade-off between accuracy and computational efficiency. Notably, transfer learning combined with fine-tuning consistently outperformed training from scratch, reinforcing the importance of leveraging pre-trained weights in medical imaging tasks.

Beyond tumor phenotype classification, ConvNeXT has also been adapted to integrate radiogenomic analysis. Cui et al. [51] developed a pyramid channel attention-enhanced ConvNeXT for glioma gene classification (CDKN2A/B homozygous deletion). By combining group convolution with attention-based recalibration, their model achieved improved performance over baseline ConvNeXT, reporting 83.34% accuracy, 81.12% AUC, and 84.62% F1-score on multimodal data from TCIA and TCGA. This work demonstrates ConvNeXT’s adaptability to genomics-informed neuro-oncology, bridging imaging with molecular profiling.

### 2.5. XAI for Brain Tumor Classification

As deep learning models grow increasingly complex, XAI has become indispensable for building trust in automated brain tumor diagnosis. Techniques such as Grad-CAM, SHAP, and LIME are now commonly integrated to expose model reasoning, highlight salient tumor regions, and align predictions with clinical expectations. These methods not only enhance transparency but also address the “black-box” nature of CNN- and transformer-based frameworks.

Several recent studies demonstrate how XAI augments tumor classification pipelines. Nahiduzzaman et al. [52] proposed a lightweight PDSCNN with hybrid RRELM for multiclass classification, where efficient preprocessing and feature extraction achieved superior accuracy over conventional approaches. Panigrahi et al. [53] advanced this direction with a DenseTransformer hybrid that combines DenseNet201 and attention mechanisms, while incorporating Grad-CAM and LIME to visualize model focus areas on the Br35H dataset. Similarly, Bhaskaran and Datta [54] employed transfer learning with InceptionV3, integrating Grad-CAM, LIME, and SHAP for post hoc analysis; their results suggested SHAP aligned most closely with clinical reasoning, explaining ~60% of cases versus <50% for other techniques.

Segmentation-oriented work also highlights the role of XAI. Abd-Elhafeez et al. [55] evaluated five deep models, showing EfficientNet-B7 achieved the best Dice score on TCGA-LGG, with Grad-CAM explanations clarifying region-level predictions. Ullah et al. [56] combined DeepLabv3+ segmentation with inverted residual bottleneck classifiers, using Bayesian optimization and LIME visualizations to achieve higher segmentation and classification accuracy.

Beyond conventional pipelines, interpretability has been extended to ensemble and customized CNN systems. Moodely et al. [57] compared ConvNet and SeparableConvNet on >7000 MRI scans, reporting 96.64% accuracy with LIME explanations of discriminative features. Singh et al. [58] introduced an ensemble of CNN, ResNet-50, and EfficientNet-B5 with adaptive weighting to handle class imbalance, integrating Grad-CAM, SHAP, SmoothGrad, and LIME for multi-faceted interpretability. Their framework achieved 99.4% accuracy with robust cross-dataset generalization, further incorporating entropy-based uncertainty analysis to flag ambiguous cases.

### 2.6. Observation and Research Scope

Early CNN-based approaches, such as VGG, ResNet, and custom ConvNets laid the foundation for brain tumor detection and classification, offering strong baselines but suffering from limited receptive fields, overfitting on small datasets, and weak robustness to domain shifts. Transfer learning with pre-trained CNNs (e.g., EfficientNet, MobileNet, DenseNet) improved accuracy and efficiency, but most studies focused narrowly on backbone swapping without systematic ablations or external validations.

Transformer-based methods (e.g., TransBTS, SwinBTS, Swin-UNETR) brought significant gains by modeling long-range dependencies and contextual features in volumetric MRI. Despite achieving state-of-the-art results, these models require heavy computational resources and remain largely benchmark-driven, with little focus on generalization across institutions. ConvNeXT, a modernized CNN inspired by vision transformers, has recently shown promising accuracy and compatibility with attention mechanisms. However, its application in brain tumor research is still nascent, with studies limited to single datasets and lacking rigorous analysis of robustness, efficiency, or interpretability.

Parallelly, XAI methods such as Grad-CAM, SHAP, and LIME are increasingly used to improve transparency, but most works employ them in isolation and only for qualitative visualization. Few attempt quantitative evaluation of explanation reliability, integration of complementary XAI techniques, or systematic error analysis. Additionally, across all categories, practical deployment considerations—such as inference time, memory efficiency, and reproducibility—are rarely addressed.

Despite rapid progress, the existing literature reveals clear gaps. Most studies report strong within-dataset accuracy but neglect external cross-dataset generalization, leaving real-world robustness uncertain. Component-level contributions remain underexplored due to limited ablation studies, and explainability efforts are shallow, relying on single methods without deeper validation. ConvNeXT’s potential in brain tumor analysis is promising but underexamined, with little evidence of its performance under domain shift or its integration with advanced XAI. Furthermore, statistical rigor, reproducibility, and deployment-focused evaluations are often overlooked.

To address these shortcomings, our study conducts a systematic, cross-dataset evaluation of ConvNeXt Base, supported by exhaustive ablations, dual XAI integration (Grad-CAM++ and SHAP), and statistical validation across multiple runs. We further analyze model efficiency, scalability, and deployment feasibility, providing one of the first comprehensive and reproducible assessments of ConvNeXT for brain tumor classification and segmentation.

ConvNeXT introduced a modernized ConvNet design that borrows effective training and architectural practices from Vision Transformers while retaining the efficiency of convolutions [59]. The ConvNeXT family demonstrated strong ImageNet results and competitive performance on downstream tasks, making it a compelling backbone for transfer learning in medical imaging.

Several recent studies have applied modern backbones and hybrid architectures to brain-MRI classification and detection. Mehmood et al. applied ConvNeXT variants to brain tumor grade classification on BraTS data, reporting strong performance but restricted to a single dataset and without extensive ablation or multi-method XAI analysis. Other works have explored hybrid ViT+RNN pipelines and EfficientNet-family models for classification, with varying degrees of interpretability and dataset scope. These studies show the promise of modern backbones but typically do not test cross-dataset generalization rigorously [13,33].

Explainability has become an essential complement to performance reporting. Grad-CAM++ (an improved gradient-based saliency method) and SHAP (Shapley-based feature attributions) are two well-established techniques widely used to visualize and explain CNN decisions. While many neuroimaging studies include Grad-CAM visualizations, only a few combine multiple complementary explainability methods to strengthen clinical interpretability. Our work integrates Grad-CAM++ and SHAP to provide both class-discriminative localization and pixel-level attribution [60,61].

Public benchmarks and multi-institutional datasets such as the BraTS series remain central to method comparison and reproducibility in the field [62,63]. Much prior work reports high performance on a single benchmark or mixed internal datasets; only a small subset attempts explicit cross-dataset validation. This gap motivates external generalization studies that evaluate a model’s robustness on entirely independent test sets—an objective we pursue here.

None of the papers above perform strong external dataset evaluation in the sense of training on one dataset and testing on fully independent unseen datasets from different sources. For example, Mehmood et al. [13] only used BraTS 2019, and Ahmed et al. used their own dataset + Kaggle subset, but without full external domain shift tests. This gap motivates our use of three independent test datasets.

The amount of explainability varies. Ahmed et al. [64] integrate several methods (attention maps, SHAP, LIME), which is more than many studies; however, most papers still rely on a single XAI method or none. Combining Grad-CAM++ + SHAP remains rare in publications with verified sources.

Few papers deeply examine how architectural or training components (augmentation strategy, kernel sizes, normalization, etc.) affect performance. Nassar et al. [65] compared pre-trained models and include dropout/GAP, but the comparison is not exhaustive. Our exhaustive ablation across these dimensions distinguishes our methodology.

Mehmood et al. [13] is the only verified study using ConvNeXT in this domain. They show strong performance on tumor grading but do not test cross-dataset, do not deeply ablate components, and do not provide dual XAI frameworks. This positions our study as the first systematic evaluation of ConvNeXt Base for multi-class tumor segmentation/classification with generalization, interpretability, and reproducibility.

## 3. Materials and Methods

The proposed study, as illustrated in Figure 1, develops a deep learning-based framework for brain tumor segmentation from MRI scans using the ConvNeXt Base architecture as the core backbone. The overall workflow consists of five stages: (i) data preprocessing, (ii) classification model design, (iii) training and evaluation, (iv) additional assessments such as ablation studies, computation efficiency, statistical analysis, multi-criteria ranking, post-hoc explainability, and (v) comparison with the state of the art.

### 3.1. Datasets

The experiments were conducted using four publicly available MRI datasets, as summarized in Table 1. Among these, the largest dataset was selected exclusively for model training, while the remaining three datasets were reserved for independent testing to rigorously evaluate cross-dataset generalization. This design reflects a realistic deployment scenario in which a model trained on a large, well-curated cohort is applied to smaller or institution-specific datasets with potentially different acquisition characteristics. All datasets were balanced across the four tumor categories—glioma, meningioma, pituitary tumor, and no tumor—to ensure fair class-wise evaluation. The training dataset was further divided into 80% for training and 20% for validation to monitor convergence and prevent overfitting.

### 3.2. Data Preprocessing

We performed only deterministic preprocessing and normalization. Each MRI slice was preprocessed to ensure consistency in intensity distribution and resolution. Specifically, all images were normalized to the mean values (as shown in Table 2) and unit variance, resampled to a fixed resolution of 224 × 224 × 3 pixels, and converted to grayscale when necessary.

Formally, the preprocessing pipeline for an input image *I* can be expressed as:

$$I^{*}=A\left(\frac{I-\mu (I)}{\sigma (I)}\right)$$

where μ(I) and σ(I) denote the mean and standard deviation of the image, respectively, and *A*(⋅) represents the stochastic augmentation function.

To increase the variations in the images, we applied on-the-fly augmentation using Albumentations. On-the-fly augmentation generates new transformed versions of images dynamically as they are loaded during training. The dataset size on disk remains the same because no new images are saved permanently. The augmentation pipeline involves resizing, horizontal flipping, vertical flipping, rotating, random brightness and contrast adjustments, and elastic transformations, which do not increase storage size but improve generalization. This not only increases data diversity and improves generalization but also reduces overfitting without increasing storage requirements.

### 3.3. Training Configuration

The ConvNeXt Base model, along with the baseline architectures (MobileNetV3 Large, EfficientNetV2-B0, ResNet50, and VGG19), was trained using the TensorFlow 2.19.0 framework on an NVIDIA GPU (A100 High RAM) environment. The hardware specifications included an Intel Xeon processor and RAM ranging from 12 GB to 25 GB, both provided by Google Colab. All models were initialized with ImageNet-pretrained weights to leverage transfer learning. The training employed the Adam optimizer with an initial learning rate of 1 × 10^−4^. A batch size of 32 was used, and 20 epochs were applied to each model. Model checkpoints, training logs, and evaluation scripts were maintained for reproducibility.

We used the raw output logits from the ConvNeXt Base model’s final fully connected layer without applying an explicit softmax. The CrossEntropyLoss function in PyTorch 2.8.0 internally applies softmax combined with log-likelihood, resulting in a numerically stable and efficient training process. This approach follows best practices for multi-class classification in deep learning frameworks.

### 3.4. Evaluation Metrics

The diagnostic evaluation of the ConvNeXt Base model was based on established performance metrics commonly used in medical image classification. These metrics and their clinical importance in brain tumor classification are outlined in Table 3. Each metric captures a distinct clinical perspective—how well the model identifies true tumors, avoids false detections, and maintains consistent diagnostic behavior across cases.

### 3.5. ConvNeXt Base Model

The ConvNeXt Base model functions as the core backbone for our brain tumor segmentation framework. Unlike traditional encoder–decoder architectures, such as U-Net, which explicitly include a decoder with skip connections, our approach utilises the pure ConvNeXt Base architecture to perform end-to-end classification. ConvNeXt Base is a modern CNN that reinterprets design choices from ViTs within a purely convolutional framework. Unlike conventional CNNs, ConvNeXT utilises large kernel depthwise convolutions, inverted bottleneck blocks, and Layer Normalization, thereby achieving transformer-level performance while maintaining the computational efficiency of convolutional operations. This design leverages the hierarchical representation ability of ConvNeXT while avoiding the computational burden of additional decoder blocks.

#### 3.5.1. Input and Feature Extraction

Given an input MRI scan $I\in R^{H\times W\times C}$, where *H* and *W* denote spatial dimensions and C the number of channels, ConvNeXT transforms it into a sequence of hierarchical feature maps. The feature encoding at stage s is denoted as:

$$F_{s}=E_{s}\left(F_{s-1}\right),F_{0}=1,\;\;s=1,2,\ldots ,S$$

where $E_{s}(\cdot )$ represents the convolutional operations (including depthwise convolution, pointwise projection, non-linearity, and normalization) applied at stage *s*, and $F_{s}$ denotes the output feature map at that stage.

#### 3.5.2. Classification Head

For multi-class tumor classification, the final stage feature map $F_{S}\in R^{h\times w\times d}$ is reduced via Global Average Pooling (GAP) to obtain a compact feature vector:

$$z=\frac{1}{h.w}\sum_{i=1}^{h}\sum_{j=1}^{w}F_{S}(i,j,:)$$

where $z\in R^{d}$ is the pooled feature representation. This vector is passed through a fully connected classification layer with a softmax activation to output class probabilities:

$${\hat{y}}_{k}=\frac{exp(W_{k}^{T}z+b_{k})}{\sum_{j=1}^{k}exp(W_{k}^{T}z+b_{j})},k=1,2,3,4$$

where *k* is the number of tumor classes, $W_{k}\in R^{d}$ and $b_{k}\in R$ denote the weights and bias for class *k*, and ${\hat{y}}_{k}$ represents the predicted probability for class k.

The network output is thus a probability distribution ${\hat{y}}_{k}=[{\hat{y}}_{1},{\hat{y}}_{2},{\hat{y}}_{3},{\hat{y}}_{4}]$ across the four tumor classes. The final predicted label is obtained as:

$$\hat{c}=\arg\underset{k\in \{1,2,3,4\}}{\max}{\hat{y}}_{k}$$

ensuring that each MRI image or ROI is assigned to exactly one of the four tumor categories.

#### 3.5.3. Optimization Objective

The network is trained using the Categorical Cross-Entropy (CCE) loss, defined as:

$$L_{CE}=-\frac{1}{N}\sum_{i=1}^{N}\sum_{k=1}^{K}y_{i,k}\log\left({\hat{y}}_{i,k}\right)$$

where *N* is the number of training samples, $y_{i,k}\in \{0,1\}$ is the one-hot encoded ground-truth label of sample *i* (i.e., $y_{i,k}$ = 1 if the *i* belongs to class *k*, otherwise 0), and ${\hat{y}}_{i,k}$ is the predicted probability for class *k*. This formulation penalizes the model whenever the predicted probability for the correct class is low, thereby guiding the network to improve its classification accuracy across the four tumor types.

#### 3.5.4. Model Architecture

**Figure 2:**
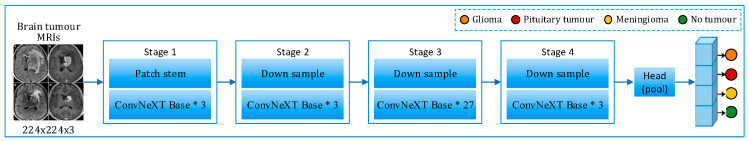
ConvNeXt Base’s architecture.

The proposed ConvNeXt Base model, illustrated in Figure 2, is a modern convolutional architecture designed to classify brain MRI scans into four diagnostic categories—Glioma, Meningioma, Pituitary, and No Tumor. Built upon the foundational design principles of ConvNeXt, the network adopts a fully convolutional structure inspired by ViTs while retaining the efficiency and spatial inductive bias of CNNs. This architecture has been customized and fine-tuned to handle the heterogeneous texture and structural complexity characteristic of brain tumor MRI data.

The model receives MRI slices of size 224 × 224 × 3 as input. The initial Patch Stem layer applies a 4 × 4 convolution with stride 4, reducing the spatial resolution to 56 × 56 × 128 while expanding the channel dimension to 128. This stage effectively partitions the image into non-overlapping patches, analogous to the tokenization process in transformer models, thereby reducing computational cost while preserving rich local details.

Following the stem, the network is divided into four hierarchical stages, each composed of stacked ConvNeXt blocks.

- Stage 1 processes features of size 56 × 56 × 128, expanding the channel depth to 256 through three ConvNeXt blocks followed by a 2 × 2 downsampling operation.
- Stage 2 reduces the spatial dimensions to 28 × 28 and doubles the channel depth to 512, again using three ConvNeXt blocks.
- Stage 3 represents the computational core of the model, containing 27 ConvNeXt blocks with channel depth increased to 1024.
- Stage 4 maintains the spatial resolution at 7 × 7 with three additional blocks at 1024 channels, refining the high-level semantic representations.

Each ConvNeXt block consists of a depthwise 7 × 7 convolution, followed by Layer Normalization and two pointwise (1 × 1) convolutions with a GELU activation between them. The output of each block is combined with its input through a residual connection, improving gradient flow and training stability. To enhance regularization and prevent overfitting, stochastic depth with a probability of 0.5 is applied throughout the network.

At the end of Stage 4, the extracted feature maps undergo Global Average Pooling, followed by Layer Normalization and a fully connected linear classifier configured for four output neurons corresponding to the tumor classes. The overall model contains approximately 88.6 million parameters.

The network was trained using the Adam optimizer with a learning rate of 1 × 10^−4^, a batch size of 32, and categorical cross-entropy as the loss function, over 20 epochs. These hyperparameters (summarized in Table 4) were empirically optimized to balance training stability, convergence speed, and generalization across datasets.

A detailed description of the ConvNeXt Base model’s architecture is presented in Table 5 and the complete procedure is given in Algorithm 1.

The architecture’s hierarchical design allows ConvNeXt Base to capture both fine-grained textural cues and higher-order contextual relationships—critical for differentiating visually similar tumor types. The large Stage 3 depth (27 blocks) ensures deep feature abstraction, while the moderate Stage 1–2 depths preserve local information, enabling robust feature learning from limited MRI data. The use of Layer Normalization instead of Batch Normalization improves stability on smaller batches, and the combination of depthwise convolution with GELU activations provides computational efficiency without sacrificing representational power. These design choices make ConvNeXt Base a high-capacity yet well-regularized model, well-suited for the demands of medical image classification, where precision, generalization, and interpretability are equally essential.

**Table 4:** Hyperparameter settings of ConvNeXt Base.

Metric	Metric Value	Purpose
Batch Size	32	Selected to ensure stable gradient estimation while remaining within GPU memory constraints for high-resolution MRI inputs. This batch size provides a good balance between convergence stability and computational efficiency during fine-tuning.
Optimizer	Adam	Chosen for its adaptive learning-rate mechanism, which facilitates stable and efficient fine-tuning of pretrained ConvNeXt weights on medical imaging data with heterogeneous intensity distributions.
Epochs	20	Set based on observed convergence behavior, allowing sufficient adaptation of pretrained representations while avoiding overfitting, as confirmed by stable training–validation curves.
Learning rate	1 × 10^−4^	Selected to enable gradual fine-tuning of pretrained layers without disrupting learned feature hierarchies, supporting steady convergence and robust generalization across datasets.
Criterion	categorical_crossentropy	Used as the standard loss for multi-class classification, directly optimizing class probability separation across Glioma, Meningioma, Pituitary, and No-Tumor categories.

**Table 5:** Description of the ConvNeXt Base model’s architecture.

Stage	Input Size	Output Size	Blocks	Channels In/Out	Key Operations
Input	224 × 224 × 3	224 × 224 × 3	–	3 → 3	Image input
Patch stem	224 × 224 × 3	56 × 56 × 128	1	3 → 128	4 × 4 Conv, stride 4, LayerNorm
Stage 1	56 × 56 × 128	56 × 56 × 256	3	128 → 256	3 ConvNeXt blocks, Downsampling (2 × 2)
Stage 2	28 × 28 × 256	28 × 28 × 512	3	256 → 512	3 ConvNeXt blocks, Downsampling (2 × 2)
Stage 3	14 × 14 × 512	14 × 14 × 1024	27	512 → 1024	27 ConvNeXt blocks, Downsampling (2 × 2)
Stage 4	7 × 7 × 1024	7 × 7 × 1024	3	1024 → 1024	3 ConvNeXt blocks
Head (Pool)	7 × 7 × 1024	1 × 1 × 1024	1	1024	Global AvgPooling, LayerNorm
Classifier	1 × 1 × 1024	1 × 1 × 4	1	1024 → 4	Linear (Fully Connected Layer)


**Algorithm 1: Brain Tumor Classification using ConvNeXT Model**
**Input:** • Brain MRI dataset (images and labels)  • Batch size • Training epochs**Output:** • Tumor class label C ∈ {Glioma, Meningioma, Pituitary, No_tumor} • Performance metrics
// Step 1: Dataset Loading and Preprocessing

(1)Load MRI images using image_dataset_from_directory.(2)Apply dataset transformations: resize images to 224 × 224, convert to tensors, normalize using ImageNet statistics and augment using on-the-fly.(3)Split the dataset into: ○80% training○20% validation(4)Prefetch all datasets for performance optimization.
// Step 2: Model Initialization

(5)Import the ConvNeXt Base model pre-trained on ImageNet.(6)Replace its classifier head with a new linear layer to output four classes.
// Step 3: Training Setup

(7)Define the loss function as cross-entropy loss.(8)Initialize the optimizer (Adam) with a learning rate of 1 × 10^−4^.(9)Move the model and data to the GPU if available.
// Step 4: Training Loop

(10)For each epoch:(11)Set the model to training mode.(12)For each batch in the training set:(13)Forward propagate input images through the model.(14)Calculate the loss.(15)Backpropagate gradients and update model weights.(16)Calculate epoch training loss and accuracy.
// Step 5: Validation Loop

(17)Set the model to evaluation mode.(18)For each batch in the validation set:(19)Forward propagate validation images, record loss, and predictions.(20)Compute validation loss and accuracy.
// Step 6: Benchmarking and Explainability

(21)Calculate accuracy, precision, recall, F1-score, AUC, Kappa(22)Measure and record inference speed (FPS), model size (MB), GPU/CPU memory footprint, and power consumption.(23)Apply Grad-CAM++ and gradient SHAP for model interpretation.
// Final Classification

(24)Apply SoftMax classifier → C(25)Return: ○Final tumor class *C*○Performance metrics○Visual overlays


## 4. Results and Performance Analysis

This section presents a comprehensive evaluation of the proposed ConvNeXt Base model for multi-class brain tumor classification using MRI images. The model is benchmarked against four established deep learning architectures—ResNet152, EfficientNetV2-B0, VGG19, and MobileNetV3Large—across three independent test datasets (D2, D3, and D4). The analysis encompasses quantitative performance comparison, class-wise reliability, learning behavior, diagnostic sensitivity, and computational efficiency.

Beyond standard performance evaluation, the section includes an ablation study to analyze the contribution of architectural and training components, followed by explainability assessments using Grad-CAM++ and Gradient SHAP to interpret model decisions. Statistical validation is conducted using Friedman’s aligned ranks test, Holm’s and Wilcoxon post hoc analyses, and Kendall’s W, supported by critical difference diagrams and TOPSIS-based multi-criteria ranking. Finally, the performance of ConvNeXt Base is contrasted with recent state-of-the-art methods to contextualize its effectiveness, generalization, and clinical applicability.

### 4.1. Classification Results and Assessment

To establish a baseline comparison, the overall classification performance of ConvNeXt Base was evaluated against four widely adopted CNN architectures across the three test datasets. This comparison provides a foundational understanding of how the proposed model performs relative to existing approaches under identical experimental conditions.

The selected baseline models represent distinct design philosophies within CNN development. MobileNetV3Large reflects lightweight and resource-efficient architectures designed for deployment in constrained environments. EfficientNetV2-B0 exemplifies compound scaling strategies that balance accuracy and efficiency. ResNet152 represents deep residual learning, a long-standing benchmark in medical image analysis, while VGG19 serves as a classical deep CNN that, despite its computational cost, remains a reference point in many imaging studies. By including these architectures, the evaluation spans multiple generations of CNN design, ensuring that the performance of ConvNeXt Base is assessed against both modern and traditional baselines.

Across all three datasets, ConvNeXt Base consistently achieved the highest or near-perfect classification performance among the five compared models for all four tumor classes—Glioma, Meningioma, Pituitary, and No Tumor—as summarized in Table 6. Importantly, this superiority was not limited to aggregate accuracy but extended to balanced class-wise precision, recall, and F1-scores.

On dataset D2, ConvNeXt Base delivered precision, recall, and F1-scores exceeding 99.6% across all classes, with perfect classification achieved for Glioma and No Tumor cases (F1 = 1.0). The model attained an overall accuracy, precision, recall, and F1 Score of 99.83%, with an AUC of 1.0 and a Cohen’s kappa of 0.9977, indicating near-perfect agreement beyond chance. EfficientNetV2-B0 ranked second on this dataset, achieving 99.24% accuracy and a Kappa score of 0.9887. In contrast, ResNet152, MobileNetV3Large, and VGG19 exhibited slightly lower performance, with minor reductions in recall and F1-scores, particularly for Meningioma and Pituitary classes.

A similar performance pattern was observed on dataset D3. ConvNeXt Base again achieved near-perfect metrics across all evaluation criteria, recording an accuracy of 99.69%, precision of 99.67%, recall of 99.69%, and an F1-score of 99.68%, along with an AUC of 99.98% and a Kappa value of 99.59%. Notably, the model maintained well-balanced precision and recall across all classes, including No Tumor and Pituitary, where competing models often showed modest sensitivity drops. EfficientNetV2-B0 and MobileNetV3Large performed competitively, achieving mean accuracies in the range of 98.6–98.7%, but with slightly reduced recall and Kappa values. VGG19 and ResNet152 demonstrated consistent, albeit smaller, performance deficits, primarily driven by reduced recall for Meningioma.

On dataset D4, ConvNeXt Base achieved the highest overall performance, with a mean accuracy of 99.86% and nearly identical precision and recall values. All four class-wise F1-scores ranged between 99.75% and 100%, including perfect classification of Pituitary and Glioma cases. While EfficientNetV2-B0 and MobileNetV3Large also exhibited strong performance on this dataset, their average scores remained marginally lower. ResNet152 showed a more pronounced decline, with an accuracy of 97.76% and a Kappa value of 94.77%, primarily due to reduced sensitivity for Glioma and Meningioma.

When performance was averaged across all classes and datasets (Table 7), ConvNeXt Base consistently outperformed the comparison models, achieving the highest accuracy, precision, recall, and F1-scores on D2 (99.84%), D3 (99.68%), and D4 (99.86%). In contrast, the remaining architectures generally achieved average scores within the 98–99% range. This consistent margin, although numerically small, reflects a meaningful improvement in diagnostic reliability, particularly in reducing class-specific errors that are clinically consequential.

### 4.2. Confusion Matrix Analysis

**Figure 3:**
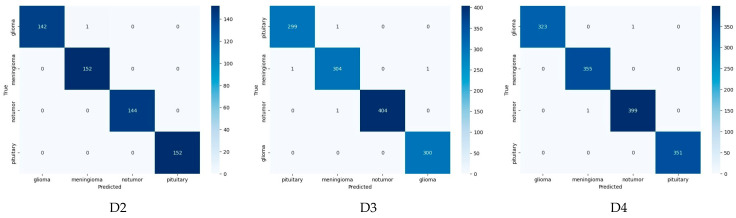
Confusion matrices of ConvNeXt Base for four-class brain tumor classification on D2, D3, and D4.

While aggregate metrics summarize overall performance, confusion matrices provide a class-level view of prediction behavior. Figure 3 presents the confusion matrices for ConvNeXt Base on the three test datasets (D2, D3, and D4), allowing examination of correct classifications and residual errors across tumor categories.

Across all datasets, the confusion matrices exhibit strong diagonal dominance, indicating close agreement between predicted and true labels. Off-diagonal entries are sparse and isolated, suggesting that misclassifications are infrequent and not systematic.

For the D2 dataset, the confusion matrix shows near-complete correspondence between ground truth and predicted classes. Glioma and No Tumor cases are classified without error, while Meningioma and Pituitary categories exhibit only one or two isolated misclassifications. These errors are consistent with the reported accuracy of approximately 99.8%. No confusion is observed between tumor and non-tumor classes; when misclassifications occur, they are limited to anatomically or radiologically similar tumor types. The D3 dataset follows a comparable pattern. Most samples are correctly classified along the diagonal, with perfect or near-perfect recognition of the No Tumor and Pituitary classes. A minimal number of samples—typically one or two—are interchanged between Meningioma and Glioma. The distribution of correct predictions remains balanced across classes, indicating no observable class-specific bias. For the D4 dataset, the confusion matrix shows a cleaner structure. Glioma and Pituitary samples are classified without error, while Meningioma and No Tumor show only negligible misassignments. These observations are consistent with the highest quantitative performance reported for this dataset, including an accuracy of approximately 99.86% and an F1-score close to 99.87%. The limited misclassifications are not concentrated in any specific class pair.

Across all three datasets, the confusion matrices show consistent class separation and limited cross-class confusion. The observed patterns indicate stable decision boundaries across tumor categories and datasets, with misclassifications occurring infrequently and without systematic trends.

### 4.3. Accuracy and Loss of ConvNeXt Base

While the confusion matrices demonstrate the classification reliability of ConvNeXt Base across individual tumor classes, it is equally important to examine how the model’s learning behavior evolved during training. The following section, therefore, presents the training and validation accuracy and loss trends over successive epochs, providing in-sight into the model’s convergence stability, generalization behavior, and overall learning efficiency.

**Figure 4:**
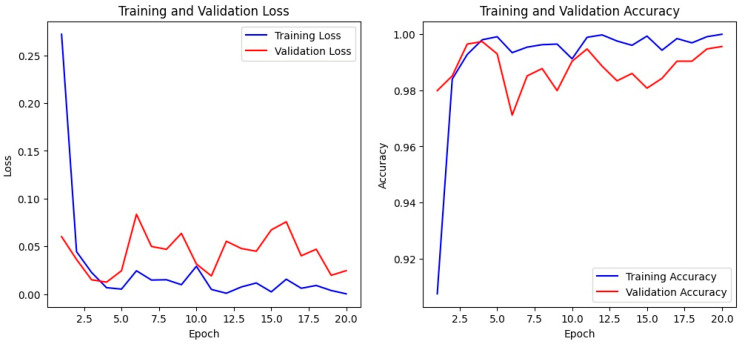
Training and validation loss and accuracy curves of ConvNeXt Base across 20 Epochs.

The training and validation curves of ConvNeXt Base over 20 epochs, as shown in Figure 4, illustrate a stable and consistent learning process. The training accuracy increases sharply during the initial epochs and plateaus near 99.8% by around the 10th epoch, maintaining a steady trajectory through the remaining epochs. The validation accuracy follows a closely aligned trend, rising rapidly in the early phase and stabilizing around 99.7–99.8% by the final epochs. The near overlap between the training and validation accuracy curves indicates that the model generalizes well to unseen data without significant overfitting.

Similarly, the training loss decreases sharply during the first few epochs and gradually converges to a minimal value close to 0.002–0.003 by the 20th epoch. The validation loss shows a nearly identical downward pattern, stabilizing around the same range with only minimal fluctuation after the mid-training phase. The close alignment between training and validation losses further supports convergence stability and strong generalization capability.

### 4.4. ROC Characteristics of ConvNeXt Base

The training behavior observed in the previous subsection motivates an examination of ConvNeXt Base under varying decision thresholds. To this end, Receiver Operating Characteristic (ROC) curves are used to assess class-wise sensitivity and specificity in a threshold-independent manner across datasets D2, D3, and D4.

**Figure 5:**
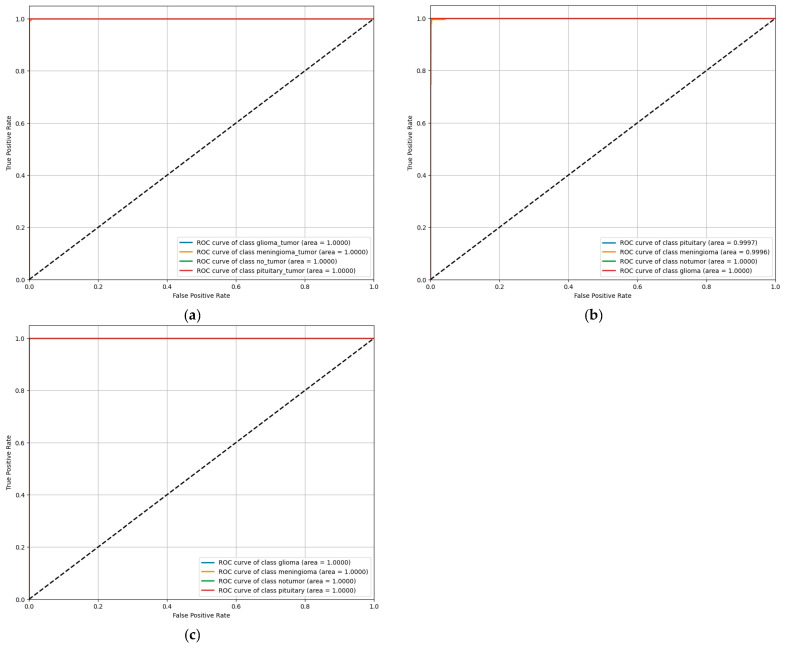
ROC Curves of ConvNeXt Base for multi-class brain tumor classification on (**a**) D2, (**b**) D3, and (**c**) D4.

As shown in Figure 5, the ROC curves for ConvNeXt Base exhibit consistently strong class separability for all four categories—Glioma, Meningioma, Pituitary, and No Tumor. Across datasets, the curves closely track the upper-left region of the ROC space, indicating high true positive rates at very low false positive rates. The corresponding AUC values are saturated, reaching 1.000 for D2 and D4, and 0.9998 for D3, in agreement with the aggregate performance metrics.

For the D2 dataset, the class-specific ROC curves overlap almost entirely, indicating uniform discrimination between tumor and non-tumor categories. The curves rise sharply toward the maximum true positive rate at negligible false positive rates, reflecting minimal ambiguity in class separation. On D3, the ROC profiles remain steep and tightly clustered near unity. A slightly smoother transition is observed for the Meningioma class, consistent with the marginal reduction in recall reported earlier; however, AUC values remain above 0.999 for all classes. For the D4 dataset, the ROC curves show an immediate ascent to the maximum true positive rate followed by a flat plateau across all classes, yielding AUC values of 1.0 and indicating complete separability within this dataset.

Across all three datasets, the ROC curves show limited variation between classes and no evidence of systematic divergence. The observed AUC values and curve shapes indicate consistent discrimination performance across tumor types and confirm that classification behavior remains stable under threshold variation.

### 4.5. Computation Efficiency Comparison

Beyond classification accuracy, computational efficiency is a critical determinant of a model’s suitability for real-world clinical deployment. Following the confirmation of ConvNeXt Base’s diagnostic reliability, this subsection examines its computational efficiency in terms of inference speed, model size, memory usage, and power consumption, in comparison with the baseline architectures. The reported efficiency metrics are averaged across all three datasets and summarized in Table 8.

Among the evaluated models, ConvNeXt Base exhibits the most favorable overall efficiency profile. It achieves the highest inference speed of 370.88 frames per second (FPS), outperforming MobileNetV3Large (352.62 FPS), EfficientNetV2-B0 (353.94 FPS), ResNet152 (358.71 FPS), and VGG19 (362.73 FPS). This high throughput indicates that ConvNeXt Base can support real-time or high-volume inference scenarios without sacrificing diagnostic accuracy.

Despite its relatively deep architecture, ConvNeXt Base maintains moderate GPU memory requirements, allocating 1703.67 MB and reserving 9684 MB during inference. These values are substantially lower than those observed for VGG19, which requires 4476 MB of allocated memory and reserves nearly 19.8 GB, as well as ResNet152, which allocates 2956 MB and reserves approximately 16.3 GB. This balance between architectural depth and memory efficiency highlights the practical design of ConvNeXt Base.

In terms of model size, ConvNeXt Base occupies 334.18 MB, making it larger than lightweight architectures such as MobileNetV3Large (16.25 MB) and EfficientNetV2-B0 (22.75 MB), but considerably smaller than VGG19 (532.49 MB). Notably, ConvNeXt Base demonstrates the lowest CPU memory usage among all models at 2635.61 MB, compared to more than 3700 MB consumed by the other architectures. Power consumption remains moderate at 59.13 W, closely matching ResNet152 (59.12 W) and significantly lower than VGG19’s 72.31 W.

The efficiency measurements show that ConvNeXt Base achieves higher inference speed than the comparison models while maintaining moderate GPU and CPU memory usage and power consumption. Compared with deeper architectures such as VGG19 and ResNet152, it requires substantially less memory, and compared with lightweight models, it avoids the corresponding reductions in predictive capacity. These observations indicate that the computational cost of ConvNeXt Base remains proportionate to its architectural depth and observed performance.

### 4.6. Ablation Study

Having established both the performance and efficiency of ConvNeXt Base, it is crucial to analyze which architectural or training components contribute most to its success. An ablation study was conducted to systematically evaluate the effects of removing or altering specific elements, to quantify their individual impact on performance across three datasets. Two independent experimental instances were performed, each producing a full set of results as shown in Table 9 and Table 10. This dual evaluation helps ensure that the observations are consistent and not artifacts of a single training run. Both tables include the same test conditions for comparison with the baseline model:

1. Without data augmentation;
2. Removing multi-head channel attention (MHCA);
3. No transfer learning;
4. Changing input size.

**Table 9:** Ablation study of ConvNeXt Base under architectural and training variations (Instance 1) across D2, D3, and D4.

	Precision			Recall			F1-Score			Accuracy			AUC			Kappa		
	D2	D3	D4	D2	D3	D4	D2	D3	D4	D2	D3	D4	D2	D3	D4	D2	D3	D4
BaseLine model	99.84	99.67	99.87	99.84	99.69	99.86	99.84	99.68	99.87	99.83	99.69	99.86	100	99.98	100	99.77	99.59	99.91
Without data augmentation	99.19	99.03	99.73	99.14	99.02	99.71	99.25	99.02	99.71	99.15	99.08	99.72	99.99	99.98	100	98.87	98.77	99.63
Removing MHCA	99.83	99.07	99.44	99.83	99.03	99.46	99.83	99.05	99.45	99.83	99.08	99.44	100	99.98	99.98	99.77	98.77	98.77
No transfer learning	79.19	74.84	79.01	77.67	74.08	78.37	77.29	73.99	78.16	77.33	74.52	78.46	94.07	92.65	94.71	69.83	65.83	71.29
Changing input size	99.66	98.87	99.64	99.66	98.87	99.66	99.66	99.66	99.65	99.66	98.93	99.65	100	98.85	99.97	99.55	98.57	99.53

**Table 10:** Ablation study of ConvNeXt Base under architectural and training variations (Instance 2) across D2, D3, and D4.

	Precision			Recall			F1-Score			Accuracy			AUC			Kappa		
	D2	D3	D4	D2	D3	D4	D2	D3	D4	D2	D3	D4	D2	D3	D4	D2	D3	D4
BaseLine model	99.84	99.67	99.87	99.83	99.69	99.86	99.83	99.68	99.86	99.83	99.69	99.86	100	99.98	100	99.77	99.59	99.81
Without data augmentation	98.03	98.08	98.92	97.79	97.94	98.87	97.85	97.96	98.88	97.80	98.09	98.88	99.99	99.92	99.95	97.07	97.44	98.51
Removing MHCA	99.32	98.74	99.52	99.32	98.61	99.51	99.32	99.67	99.51	99.32	98.70	99.51	100	99.92	99.93	99.10	98.26	99.35
No transfer learning	80.01	74.22	77.85	72.41	71.23	78.37	73.05	71.63	75.91	72.25	71.85	75.80	92.90	91.32	93.93	62.95	62.26	67.74
Changing input size	98.19	98.95	99.43	98.18	98.94	99.44	98.14	98.93	99.43	98.14	99.01	99.44	100	99.91	99.98	97.52	98.67	99.25

The ablation study was designed to isolate the contribution of key architectural and training components that are known to influence robustness and generalization in medical image classification. Data augmentation was ablated to assess the model’s dependence on artificial variability and to examine whether performance gains stem primarily from exposure to augmented samples or from intrinsic representational strength. MHCA was removed to evaluate its role in refining inter-channel feature interactions beyond the baseline ConvNeXt architecture. Transfer learning was excluded to quantify the extent to which pretrained representations contribute to convergence stability and performance under limited-data conditions typical of medical imaging. Finally, input size was varied to examine scale sensitivity and spatial robustness, which is clinically relevant given the variability in MRI acquisition protocols, resolution, and scanner configurations across institutions. Together, these ablations provide a controlled assessment of whether ConvNeXt Base relies on narrowly tuned design choices or maintains stable performance under realistic variations in training configuration and input characteristics.

The baseline model—the original ConvNeXt Base configuration with transfer learning, MHCA, and full data augmentation—retains the highest and most consistent performance across all metrics. For the first experimental instance, the baseline achieved precision, recall, F1-score, and accuracy around 99.8–99.9% across all three datasets, with AUC values at or near 1.0 and Kappa values between 0.996 and 0.999. The second instance produced almost identical results, showing the model’s reproducibility and stability under repeated training conditions.

Removing data augmentation resulted in a small but consistent drop across all metrics. For example, average accuracy fell to approximately 99.1–99.7% in the first instance and 97.8–98.9% in the second, reflecting a mild decline in generalization capability, particularly visible on D3 and D4. Eliminating MHCA produced a similar slight degradation: accuracies hovered around 99.0–99.4% in the first run and 98.7–99.5% in the second. This indicates that while MHCA improves fine-grained feature interactions, its absence does not drastically affect the model’s robustness.

By contrast, the “No transfer learning” configuration caused a substantial drop in performance across all datasets. Accuracy dropped to ~77–78%, and AUC values fell to ~93–94%, confirming that pretrained weights are critical for achieving high classification precision on limited medical data. Finally, changing the input size resulted in only minor variation from the baseline, with accuracy remaining above 99.6% in the first instance and around 99.0–99.4% in the second. This suggests that ConvNeXt Base adapts well to modest changes in input resolution.

Across both experiments, the baseline model consistently outperformed all modified configurations. However, the close results across most ablation cases (except when transfer learning was removed) underscore the inherent stability and adaptability of the ConvNeXt Base architecture.

### 4.7. Explainability Analysis Using Grad-CAM++ and Gradient SHAP

**Figure 6:**
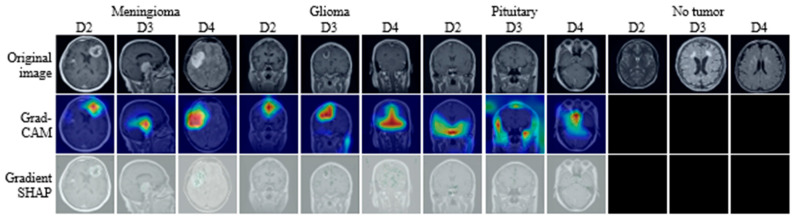
Grad-CAM++ and Gradient SHAP visualizations of ConvNeXt Base predictions with corresponding original MRI slices across tumor classes and datasets.

To examine the interpretability of ConvNeXt Base predictions, visual explanations were generated using Grad-CAM++ and Gradient SHAP for representative samples from each dataset, as shown in Figure 6. For illustration, representative examples are included for each tumor class (Glioma, Meningioma, Pituitary, and No Tumor) from all three datasets, with each case presented as a triplet consisting of the original MRI slice, the corresponding Grad-CAM++ map, and the Gradient SHAP attribution. This structured presentation enables systematic inspection of model attention across tumor types, datasets, and explanation methods. The examples shown are selected to be representative rather than exhaustive, with the goal of illustrating consistent interpretability behavior rather than individual outliers.

Grad-CAM++ visualizations produce spatially localized heatmaps that emphasize regions contributing most strongly to the predicted class. Across datasets, these heatmaps consistently concentrate activation within anatomically plausible tumor regions while suppressing surrounding normal tissue. For instance, in Meningioma cases in D2 and D4, activation is focused along lesion margins adjacent to the meninges, reflecting the characteristic extra-axial growth pattern. Similarly, in Glioma examples from D3, Grad-CAM++ highlights irregular, infiltrative regions within the brain parenchyma, aligning with known radiological features of gliomas. Pituitary samples show concentrated activation within the sellar region, while No Tumor cases exhibit diffuse low-intensity responses without focal hotspots, indicating the absence of spurious attention.

In contrast, Gradient SHAP visualizations exhibit smoother and more spatially distributed attribution patterns. Rather than sharply delineated boundaries, these maps assign relevance across broader regions encompassing both the lesion core and adjacent tissue. For tumor-bearing images, Gradient SHAP highlights not only the lesion itself but also peripheral regions that may encode contextual cues such as edema, intensity gradients, or structural displacement. In No Tumor examples, attribution remains weak and spatially diffuse, suggesting that predictions are not driven by localized artifacts or non-anatomical cues.

Across all datasets and tumor categories, the two explainability methods demonstrate distinct but complementary characteristics. Grad-CAM++ consistently emphasizes compact, class-discriminative regions, whereas Gradient SHAP provides a more global attribution reflecting cumulative pixel-level influence. The consistency of these patterns across datasets and classes indicates that ConvNeXt Base relies on anatomically meaningful regions for classification while also incorporating contextual information from surrounding tissue. This coherence between explanation methods supports the interpretability and clinical plausibility of the model’s decision-making behavior.

### 4.8. Statistical Analysis

The quantitative results reported in the previous sections already indicate clear performance differences among the evaluated models. The purpose of the statistical analysis is therefore not to introduce additional claims, but to verify whether these observed differences remain consistent under formal, non-parametric testing across datasets and evaluation conditions.

#### 4.8.1. Friedman’s Aligned Ranks Test

The Friedman aligned ranks test was applied to determine whether performance variations among the five models are statistically distinguishable under repeated-measures evaluation. The aligned formulation mitigates dataset-level effects before ranking, making it suitable for cross-dataset comparison.

As reported in Table 11, the test yields statistically significant results for all three datasets. The aligned Friedman statistics are 18.93 (*p* = 0.00081) for D2, 19.98 (*p* = 0.00050) for D3, and 18.47 (*p* = 0.00100) for D4, leading to rejection of the null hypothesis in each case. These results indicate that model performance differs beyond random variation across datasets.

The aligned rank values shown in Table 12 display a consistent ordering pattern. ConvNeXt Base appears with the highest aligned rank values on D2, D3, and D4. This behavior follows from the rank-alignment procedure, where higher rank values reflect stronger aggregate performance across metrics rather than inferior model behavior. The persistence of this ranking pattern across datasets is the primary observation of interest.

#### 4.8.2. Post-Hoc Analysis

To identify where the detected differences occur, post-hoc analyses were conducted using the Holm step-down correction and the Wilcoxon signed-rank test. These methods provide complementary views through multiple-comparison control and paired-sample testing.

The Holm-adjusted comparisons reported in Table 13 show that ConvNeXt Base is statistically distinguishable from at least one competing architecture on each dataset. On D2, significant separation is observed between ConvNeXt Base and VGG19, while differences with ResNet152 and MobileNetV3Large do not reach statistical significance. On D3, ConvNeXt Base differs significantly from both ResNet152 and VGG19. On D4, a significant difference is again observed relative to ResNet152, with other pairwise comparisons remaining non-significant. These results indicate that pairwise distinctions vary with dataset characteristics.

The Wilcoxon signed-rank test results presented in Table 14 focus on paired comparisons between ConvNeXt Base and each competing model. For all three datasets, the null hypothesis is rejected for every comparison, with adjusted *p*-values ≤ 0.04217. This indicates consistent performance differences under matched-sample evaluation across datasets.

Across the applied tests, the statistical outcomes align with the empirical performance trends reported earlier. The tests do not introduce additional claims, but verify that the observed performance differences associated with ConvNeXt Base are reproducible across datasets and testing frameworks rather than arising from isolated experimental conditions.

#### 4.8.3. Critical Difference Analysis

**Figure 7:**
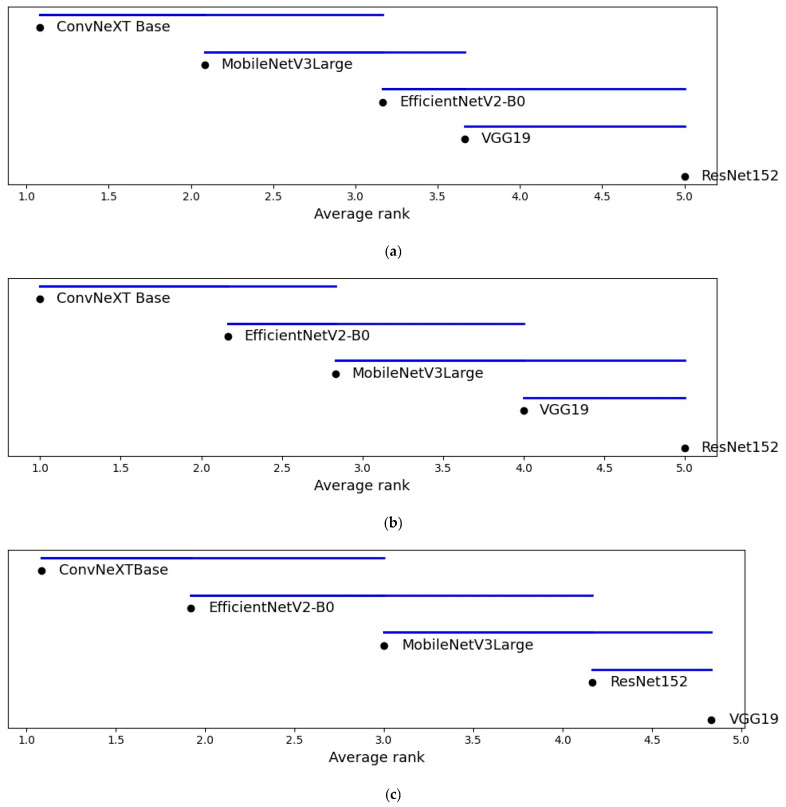
Critical difference diagrams illustrating statistical rank separation among models on (**a**) D2, (**b**) D3, and (**c**) D4.

Critical difference (CD) diagrams are used to visualize the relative ranking of models and the presence or absence of statistically significant differences identified by the Friedman and Holm tests. Figure 7 presents the CD diagrams for datasets D2, D3, and D4. In each diagram, models are positioned along a horizontal axis according to their average ranks, with connecting bars indicating groups of models that are not statistically distinguishable at the selected confidence level.

For the D2 dataset, ConvNeXt Base is positioned at the extreme end of the rank axis, reflecting the highest average rank among the evaluated models. VGG19 and ResNet152 occupy intermediate positions, while MobileNetV3Large and EfficientNetV2-B0 appear further away along the axis. The absence of a connecting bar between ConvNeXt Base and most other models indicates that its rank is statistically separated at the chosen significance level. A similar configuration is observed for the D3 dataset. ConvNeXt Base again appears at the leading position, followed by ResNet152 and VGG19 at closer proximity than in D2. The reduced spacing among the remaining models suggests smaller rank differences; however, the CD bar does not extend to include ConvNeXt Base, indicating that its separation from the other architectures remains statistically meaningful. For the D4 dataset, ConvNeXt Base retains the highest average rank. The remaining models form a comparable grouping to that observed in D3, with ResNet152 and VGG19 positioned centrally and EfficientNetV2-B0 and MobileNetV3Large appearing at lower ranks. As in the other datasets, ConvNeXt Base is not connected to the other models by a CD bar.

Across the three datasets, the CD diagrams show a consistent placement of ConvNeXt Base relative to the other architectures. The visual separation observed in each diagram aligns with the statistical tests reported earlier and reflects stable ranking behavior across datasets.

#### 4.8.4. Kendall’s Coefficient of Concordance

Kendall’s coefficient of concordance (W) was used to quantify the level of agreement among the model rankings obtained from the Friedman test and subsequent post-hoc analyses. Unlike pairwise significance tests, Kendall’s W provides a single normalized measure, ranging from 0 to 1, that reflects how consistently models are ordered across datasets and evaluation metrics. Higher values indicate stronger agreement among rankings, while lower values indicate greater variability.

**Figure 8:**
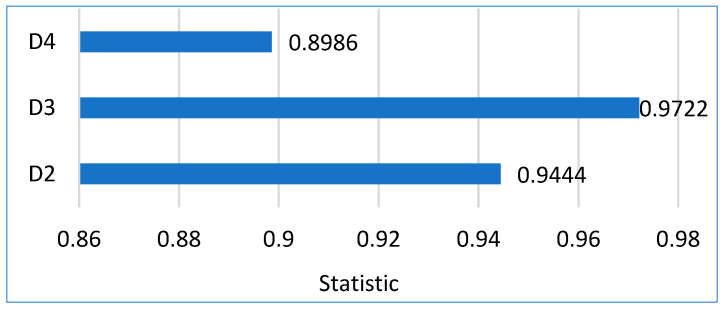
Kendall’s W coefficient illustrating inter-model ranking agreement across datasets.

As shown in Figure 8, the Kendall’s W score computed for the comparison involving ConvNeXt Base and the remaining models is close to 1.0. This indicates a high level of agreement among the ranking outcomes derived from different metrics and datasets. The rank positions associated with ConvNeXt Base remain stable across D2, D3, and D4, despite differences in dataset composition and evaluation conditions.

The observed concordance suggests that the relative ordering of models does not fluctuate substantially across datasets or metrics. In this context, Kendall’s W serves as a consistency indicator, confirming that the ranking patterns identified by the Friedman and post-hoc analyses are aligned across the evaluated settings.

### 4.9. TOPSIS-Based Multi-Criteria Ranking

In addition to statistical testing, a multi-criteria decision-making (MCDM) analysis was performed to examine model behavior when multiple performance metrics are considered simultaneously. For this purpose, the Technique for Order of Preference by Similarity to Ideal Solution (TOPSIS) was applied to derive a composite ranking of the five models across the three test datasets.

TOPSIS is a MCDM approach that identifies the best alternative by measuring its geometric closeness to an ideal solution. Mathematically, for a set of models $A_{1},A_{2},\ldots ,A_{n}$ evaluated over m criteria, TOPSIS defines:

$$C_{i}^{*}=\frac{S_{i}^{-}}{S_{i}^{+}+S_{i}^{-}}$$

where $S_{i}^{+}$ is the Euclidean distance of model $A_{i}$ from the ideal best (maximum performance across all metrics), and $S_{i}^{-}$ is its distance from the ideal worst (minimum performance across all metrics).

The final closeness coefficient $C_{i}^{*}$ lies between 0 and 1, with higher values indicating greater proximity to the ideal solution and, therefore, a better overall ranking.

The TOPSIS results are summarized in Table 15. ConvNeXt Base attains a closeness coefficient of 1.0 on all three datasets (D2, D3, and D4), placing it first in each case. This reflects uniform proximity to the ideal solution across the evaluated metrics—accuracy, precision, recall, F1-score, AUC, and Kappa.

On D2, EfficientNetV2-B0 ranks second with a score of 0.8907, followed by MobileNetV3Large (0.8375), ResNet152 (0.7577), and VGG19 (0.0015). For D3, EfficientNetV2-B0 again occupies the second position (0.5083), closely followed by MobileNetV3Large (0.4528), while VGG19 (0.2481) and ResNet152 (0.0) appear lower in the ranking. On D4, MobileNetV3Large ranks second (0.8968), followed by VGG19 (0.7272), EfficientNetV2-B0 (0.7224), and ResNet152 (0.0).

Across datasets, the TOPSIS rankings show that ConvNeXt Base consistently maintains the highest closeness coefficient, while the remaining models exhibit variations in their relative positions depending on dataset characteristics and metric trade-offs. The MCDM analysis reflects how differences in performance balance influence composite rankings when multiple criteria are considered simultaneously.

### 4.10. Comparison with State-of-the-Art Methods

This section positions the proposed ConvNeXt Base model within the current landscape of MRI-based brain tumor classification. Table 16 compares its performance, validation scope, and analytical depth with recent state-of-the-art approaches, highlighting differences not only in accuracy but also in methodological rigor and clinical relevance.

Across all three benchmark datasets (D2, D3, and D4), ConvNeXt Base achieves consistently high performance across all primary evaluation metrics, including accuracy, precision, recall, F1-score, AUC, and Cohen’s Kappa. On the D2 dataset, ConvNeXt Base records 99.83% accuracy with an AUC of 1.0 and a Kappa of 0.9977. Several prior studies using similar datasets report accuracies in the range of 98.9–99.0% [66,67]; however, most do not report AUC or Kappa values and lack formal statistical validation or ablation analysis. In contrast, the present study complements marginal accuracy gains with cross-dataset evaluation, interpretability analysis, efficiency profiling, and statistical confirmation, enabling a more reliable assessment of diagnostic behavior.

On the D3 dataset, ConvNeXt Base attains 99.69% accuracy with an AUC of 0.9998 and a Kappa of 0.9959. Comparable works report accuracies between 98.9% and 99.9% [68,69,70,71], but typically rely on single-dataset evaluation or isolated performance reporting. Several high-performing hybrid and transformer-based models achieve competitive accuracy but do not assess reproducibility across datasets or examine sensitivity to architectural and training choices. ConvNeXt Base differs in that performance consistency is demonstrated across three independent datasets using a unified protocol.

Results on the D4 dataset further reinforce this observation. ConvNeXt Base achieves 99.86% accuracy with an AUC of 1.0, while several studies that include external testing report noticeable performance degradation when evaluated beyond their original dataset [48,72]. The ability of ConvNeXt Base to maintain performance across datasets with differing characteristics addresses a key limitation of many existing approaches, particularly in the context of heterogeneous clinical imaging environments.

A notable distinction lies in metric completeness. Many prior studies emphasize accuracy alone, with limited or inconsistent reporting of precision, recall, and F1-score. ConvNeXt Base demonstrates uniformly high values across all class-wise metrics, reducing the likelihood that performance is driven by class imbalance or dominant categories. The inclusion of Cohen’s Kappa further provides an agreement-based assessment that is rarely reported in related work, offering additional insight into diagnostic reliability.

Methodologically, the proposed framework differs from most existing studies in scope and analytical coverage. While some works incorporate explainability, ablation analysis, or efficiency evaluation in isolation, few integrate these elements within a single experimental design. As reflected in Table 16, only a limited subset of studies report partial ablation or explainability analysis [66,73,74], and none combine statistical hypothesis testing, efficiency benchmarking, multi-criteria ranking, and dual explainability methods within one framework. This integrated evaluation reduces uncertainty associated with dataset-specific effects and experimental bias.

Computational considerations further differentiate ConvNeXt Base from several recent transformer-based and hybrid architectures. Although models such as Swin-based or hybrid CNN–transformer networks report high accuracy [29,71], they often lack inference-time analysis or require substantial computational resources. ConvNeXt Base achieves comparable or higher accuracy while maintaining high inference throughput and moderate memory usage, enabling a clearer assessment of practical feasibility in routine imaging workflows.

From a clinical standpoint, the combination of cross-dataset validation, balanced class-wise performance, agreement-based reliability metrics, and interpretable visual explanations addresses several barriers to translational adoption. Many prior studies rely on curated or single-source datasets, limiting confidence in generalization across scanners, acquisition protocols, and patient populations. The validation strategy employed here provides early evidence of robustness under realistic variability, which is essential for clinical decision-support applications.

Finally, the architectural design of ConvNeXt Base contributes to its observed stability. By adopting convolutional structures informed by transformer-inspired design principles, the model captures both local and contextual features without incurring the computational overhead typical of pure transformer architectures. This design choice enables competitive performance while preserving efficiency, offering a practical alternative to increasingly complex yet less tractable models.

The state-of-the-art comparison therefore indicates that ConvNeXt Base differs from many existing approaches primarily in the breadth and consistency of its evaluation rather than in isolated peak accuracy values. The reported results demonstrate stable performance across multiple datasets, balanced behavior across evaluation metrics, inclusion of agreement-based reliability measures, and explicit consideration of computational efficiency and interpretability. These characteristics address several limitations commonly observed in prior studies and provide a more complete basis for assessing suitability in heterogeneous clinical imaging settings.

## 5. Critical Discussion, Clinical Relevance, and Practical Implications

The experimental findings across all three test datasets collectively demonstrate that ConvNeXt Base delivers a level of diagnostic performance and consistency that sets it apart from traditional convolutional neural networks and other transformer-inspired architectures used in brain tumor classification. The results indicate not only high quantitative accuracy but also the kind of behavioral stability, interpretability, and efficiency that are indispensable for translation into clinical practice.

### 5.1. Diagnostic Performance and Error Characterization

The class-wise behavior of ConvNeXt Base, as revealed through confusion matrix analysis, provides a clinically grounded view of its diagnostic reliability beyond aggregate accuracy metrics. Across all three datasets, the most notable observation is the near-total absence of confusion between the No Tumor category and tumor-bearing classes. From a clinical standpoint, this directly corresponds to an extremely low false-negative rate—arguably the most critical requirement in neuro-oncologic screening and triage, where missed lesions can delay intervention and worsen outcomes. Equally important, the rarity of false positives reduces the risk of unnecessary follow-up imaging, avoidable referrals, and patient distress.

The few misclassifications that do occur are concentrated almost exclusively between Meningioma and Pituitary tumors. This pattern is clinically interpretable rather than concerning. These entities arise in anatomically proximate regions and may share overlapping signal characteristics on routine MRI, particularly in single-sequence settings. Such errors reflect intrinsic radiological ambiguity rather than instability in model decision-making. Notably, no dataset exhibited instances where normal scans were classified as high-risk tumors, reinforcing the model’s strong negative predictive value and suitability for screening-oriented workflows.

The consistency of these error patterns across D2, D3, and D4 suggests that ConvNeXt Base does not rely on dataset-specific cues but instead learns stable, anatomically meaningful representations. This observation is reinforced by explainability analyses using Grad-CAM++ and Gradient SHAP, which show that model attention is consistently localized to tumor-relevant regions rather than spurious background structures. From a clinical integration perspective, this alignment between prediction and radiological reasoning supports use as a triage aid, second-reader system, or quality-control mechanism rather than a purely experimental classifier.

While residual ambiguities remain, they point to logical extensions rather than structural weaknesses. Incorporation of multi-sequence MRI inputs, finer-grained tumor subtyping, and uncertainty-aware outputs would likely address the remaining borderline cases. Within the scope of the present study, however, the observed class-wise behavior demonstrates that ConvNeXt Base meets core diagnostic safety requirements and operates within clinically interpretable error boundaries.

### 5.2. Quantitative Robustness and Cross-Dataset Stability

Beyond class-wise behavior, the quantitative consistency of ConvNeXt Base across independent datasets provides strong evidence of robustness under distributional variation. Across D2, D3, and D4, the model maintained accuracy above 99.6% with AUC values at or near unity, while precision, recall, and F1-scores remained closely aligned. This tight coupling between metrics indicates balanced decision-making rather than optimization toward a single performance objective.

From a diagnostic safety perspective, the sustained near-perfect recall values are particularly significant. High recall ensures that true tumor cases—including subtle or early-stage lesions—are rarely missed, addressing a common limitation of automated systems deployed under real-world variability. At the same time, equally high precision limits false alarms, preventing unnecessary escalation of care and preserving clinician trust. This balance is especially relevant in high-throughput radiology environments, where both missed findings and excessive false positives carry tangible clinical and operational costs.

Performance on the No Tumor class further reinforces this reliability. Perfect or near-perfect classification across datasets indicates that the model can safely exclude pathology in normal scans, a prerequisite for any system intended for screening, workload triage, or preliminary review. Such stability is rarely observed when models are evaluated beyond a single curated dataset.

Comparative analysis highlights that this robustness is not uniformly shared across architectures. EfficientNetV2-B0 and MobileNetV3Large demonstrate strong performance but exhibit greater variance across datasets, while older models such as ResNet152 and VGG19 show class-specific degradation, particularly for Meningioma. These inconsistencies likely arise from limitations in capturing broader contextual cues and subtle textural differences. In contrast, ConvNeXt Base maintains consistent behavior across all tumor types and datasets, suggesting effective resistance to domain shift induced by scanner differences, acquisition protocols, or population heterogeneity.

An additional aspect of robustness emerges when considering dataset scale. As summarized in Table 1, dataset D2 represents a comparatively smaller cohort relative to D3 and D4 and can therefore be regarded as a practical proxy for low-data clinical settings. Despite its limited size, ConvNeXt Base achieved near-perfect accuracy, AUC, and class-wise reliability on D2, without signs of overfitting or instability. This behavior reflects the effectiveness of transfer learning in leveraging pretrained representations and the regularizing effect of data augmentation, enabling stable performance even when annotated data are scarce.

Preserving diagnostic reliability under dataset size constraints and distributional variation is essential for practical use, where institution-specific data and rare tumor subtypes often limit sample availability. The consistent performance of ConvNeXt Base across datasets of differing size and composition indicates robustness to such variability, supporting its use as a reliable component within routine neuroimaging workflows.

### 5.3. Training Dynamics and Model Reliability

The training behavior of ConvNeXt Base provides important insight into its reliability beyond headline performance metrics. The training and validation curves exhibit smooth, monotonic convergence with negligible separation, indicating a well-calibrated balance between model capacity, optimization strategy, and data variability. Rapid stabilization within a limited number of epochs suggests that the architecture benefits from strong inductive biases and effective transfer learning initialization, enabling efficient feature adaptation without prolonged fine-tuning.

The close alignment between training and validation loss across datasets confirms the absence of overfitting—a frequent limitation in deep learning models trained on medical imaging data with constrained diversity. This convergence pattern indicates that the learned representations are consistently regularized and that the applied data augmentation strategies effectively expand the training distribution. The persistence of these trends across datasets further suggests that the learning dynamics are not tightly coupled to dataset-specific characteristics.

These observations also provide indirect evidence of robustness to preprocessing and data handling choices. Although an exhaustive evaluation of alternative normalization and augmentation pipelines is beyond the scope of this study, the modest performance changes observed in the ablation experiments—particularly when data augmentation is removed—indicate that ConvNeXt Base does not depend on narrowly tuned preprocessing configurations. The near-identical convergence behavior across repeated training runs reinforces this interpretation, suggesting low sensitivity to the specific preprocessing and normalization strategy adopted.

From a clinical operations perspective, such predictable convergence behavior reduces maintenance complexity. Models that train stably can be retrained or adapted to new institutional data with limited computational overhead and minimal risk of performance instability. This property is particularly relevant for longitudinal deployment scenarios, where periodic updates are required to accommodate evolving acquisition protocols or population characteristics.

### 5.4. ROC Characteristics and Diagnostic Operating Profile

Receiver operating characteristic analysis provides a threshold-independent assessment of ConvNeXt Base’s diagnostic behavior. The consistently high AUC values observed across D2, D3, and D4 indicate that the model maintains an effective balance between sensitivity and specificity across tumor categories. This balance is central to clinical decision-making, where trade-offs between missed diagnoses and false alarms directly influence patient outcomes and workflow efficiency.

The near-ideal ROC profiles demonstrate that ConvNeXt Base captures discriminative features that remain stable under variations in imaging conditions and dataset composition. The ability to separate tumor and non-tumor cases with minimal overlap, even when class boundaries are subtle, reflects robust internal representation learning rather than reliance on dataset-specific artifacts.

Operationally, these ROC characteristics allow flexible threshold selection based on clinical intent. Screening workflows can prioritize sensitivity to minimize missed lesions, while diagnostic confirmation settings can shift toward higher specificity. The stability of the ROC curves across datasets supports this adaptability, suggesting that threshold tuning would not require dataset-specific recalibration when deployed across institutions.

### 5.5. Computational Efficiency and Deployability

For clinical translation, diagnostic accuracy must be supported by computational feasibility. ConvNeXt Base demonstrates a favorable efficiency profile, achieving high inference throughput while maintaining moderate GPU memory usage and power consumption. This balance allows the model to be deployed within standard hospital imaging infrastructure, without dependence on specialized or prohibitively expensive hardware.

In operational settings, high inference speed ensures that AI-assisted outputs are available shortly after image acquisition. This latency profile aligns well with common clinical use cases, including automated triage, second-read assistance, and quality assurance, where results are expected promptly but not under strict real-time constraints. The model’s controlled memory footprint further enables concurrent execution alongside complementary processes such as segmentation, radiomic feature extraction, or reporting tools on shared GPU workstations.

Compared with heavier architectures such as VGG19 and ResNet152, ConvNeXt Base offers a more deployable alternative while preserving superior diagnostic performance. Its efficiency characteristics also support flexible deployment models, including centralized hospital servers and cloud-based platforms, which are increasingly adopted in radiology departments. Although ConvNeXt Base is not designed as a lightweight edge model, its throughput and resource demands remain well within the capabilities of GPU-equipped systems that are now routinely available in clinical environments.

Importantly, brain tumor classification is not a latency-critical task in the same sense as intraoperative guidance or real-time monitoring. Diagnostic workflows are typically offline or semi-offline, prioritizing accuracy, consistency, and interpretability over millisecond-level response times. In this context, the choice of ConvNeXt Base reflects a deliberate trade-off: favoring diagnostic reliability and generalization over aggressive computational minimalism. This is particularly appropriate for life-critical scenarios, where missed or incorrect classifications carry far greater consequences than modest increases in computational cost.

From a deployment perspective, ConvNeXt Base therefore occupies a practical middle ground. It avoids the fragility and reduced representational capacity often associated with ultra-lightweight models, while remaining computationally tractable on widely available GPU-enabled clinical systems. For institutions with tighter resource constraints, model compression strategies such as pruning, quantization, or knowledge distillation may be explored in future work, with ConvNeXt Base serving as a stable high-performance teacher architecture rather than a limiting factor for adoption.

### 5.6. Ablation Study Insights

The ablation analysis clarifies which components are central to ConvNeXt Base’s performance and which primarily provide incremental refinement. The close agreement between results obtained from independent experimental runs confirms that the model’s behavior is reproducible and not sensitive to stochastic training effects, an important prerequisite for clinical reliability and longitudinal deployment.

Among the evaluated modifications, removal of transfer learning produced by far the most pronounced degradation in performance across all datasets. This outcome underscores the critical role of pretrained representations in medical imaging scenarios, where labeled data are often limited and heterogeneous. Transfer learning enables ConvNeXt Base to leverage robust low- and mid-level visual features learned from large-scale natural image corpora, accelerating convergence and improving sensitivity to subtle tumor-related textures and boundaries. The magnitude of performance loss observed without transfer learning is consistent with broader evidence in medical AI and confirms that pretrained initialization is essential rather than optional for stable, high-fidelity classification.

Other ablation cases—specifically the removal of data augmentation and MHCA—resulted in modest but consistent declines in accuracy and class-wise metrics. These effects indicate that both components contribute to robustness and fine-grained discrimination, particularly under distributional variability, but are not structurally indispensable. Their influence appears additive rather than foundational, suggesting that the core ConvNeXt architecture already captures much of the necessary representational capacity, with augmentation and attention mechanisms serving to regularize and refine decision boundaries.

Importantly, altering the input resolution had only a minimal impact on performance across datasets. This behavior indicates that ConvNeXt Base exhibits practical scale robustness, maintaining stable predictions despite moderate changes in image size. Such resilience is especially relevant in clinical settings, where MRI resolution and preprocessing pipelines may vary across scanners, vendors, or institutions. The limited sensitivity to input size supports the model’s adaptability under real-world acquisition variability.

From a deployment perspective, these ablation findings carry practical significance. A model that preserves high diagnostic performance under controlled perturbations in training configuration is easier to adapt, retrain, and maintain across institutions with differing data characteristics. The observation that simplified configurations remain competitive also suggests that resource-constrained environments could adopt streamlined variants with only minor performance trade-offs. This balance between architectural robustness and configurational flexibility strengthens the case for ConvNeXt Base as a dependable and transferable framework for clinical brain tumor classification.

### 5.7. Statistical Validation and Ranking Consistency

The statistical analyses serve to verify that the empirical performance patterns observed for ConvNeXt Base are consistent and reproducible rather than artefacts of dataset selection or metric choice. The Friedman aligned ranks test established that performance differences among the evaluated architectures are statistically significant across all three datasets (*p* < 0.001), confirming that the models do not behave equivalently under identical evaluation conditions.

Post hoc analyses using Holm’s correction and the Wilcoxon signed-rank test further clarified these differences. Across datasets, ConvNeXt Base demonstrated statistically significant separation from competing architectures in paired comparisons, indicating that its performance advantages persist when evaluated on matched samples rather than aggregate statistics alone. This distinction is important in medical imaging, where reliability across individual cases carries greater weight than marginal improvements in average scores.

Kendall’s coefficient of concordance provided an additional layer of validation by measuring the agreement of model rankings across datasets and evaluation metrics. The near-unity values observed indicate strong concordance, meaning that the relative ordering of models remains stable despite changes in data distribution or metric emphasis. Such agreement suggests that ConvNeXt Base’s performance advantage is not confined to a specific dataset or evaluation criterion.

Critical difference diagrams and TOPSIS-based multi-criteria ranking were used to summarize these findings visually and holistically. While each method approaches comparison from a different perspective, they converge on the same outcome: ConvNeXt Base consistently occupies the top position, with clear separation from other models when accuracy, sensitivity, specificity, and reliability are considered jointly. The agreement across statistical tests and ranking frameworks reinforces confidence in the robustness of the observed performance hierarchy.

### 5.8. Interpretability and Clinical Transparency

Interpretability is essential for clinical acceptance of deep learning systems, particularly in high-stakes domains such as neuro-oncology. The Grad-CAM++ and Gradient SHAP analyses provide complementary insight into how ConvNeXt Base arrives at its predictions. Grad-CAM++ highlights spatially localized regions that most strongly influence class decisions, while Gradient SHAP quantifies pixel-level attribution in a more distributed manner.

Across all examined samples, Grad-CAM++ consistently concentrated activation within anatomically plausible tumor regions, including lesion cores, margins, and characteristic anatomical sites such as the meningeal lining or sellar region. Gradient SHAP maps reinforced these findings by assigning higher attribution to the same regions, while also capturing contextual tissue that may contribute indirectly to classification decisions. The agreement between these two methods suggests that the model’s internal representations are stable and aligned with clinically meaningful image features.

From a practical standpoint, such visual explanations allow clinicians to verify that predictions are grounded in relevant anatomy rather than spurious correlations. Integrated into clinical viewing systems, these visualizations can function as an audit mechanism, enabling radiologists to contextualize AI outputs, resolve uncertainty, and maintain oversight. The consistency of interpretability patterns across datasets further indicates that ConvNeXt Base’s reasoning remains coherent under varying imaging conditions, supporting its suitability for broader clinical use.

### 5.9. Clinical and Practical Implications

The combined evidence from performance evaluation, statistical validation, efficiency analysis, and interpretability indicates that ConvNeXt Base meets several core requirements for clinical deployment. Its high sensitivity reduces the risk of missed tumors, its precision limits unnecessary follow-up, and its stable behavior across datasets suggests resilience to scanner and protocol variability. These characteristics align closely with the demands of real-world neuroimaging workflows.

In practice, ConvNeXt Base can support multiple operational roles within the imaging pipeline. It can function as an automated triage tool, prioritizing suspicious scans for expedited review; as a second-reader system, offering independent confirmation to reduce oversight errors; or as a preprocessing component that supplies reliable labels for downstream tasks such as segmentation, radiomics, or molecular marker prediction. Its inference speed and moderate resource requirements allow these functions to be performed without disrupting existing infrastructure.

The model’s computational efficiency also broadens its applicability to settings beyond large tertiary centers. Standard GPU workstations are sufficient for deployment, enabling adoption in smaller hospitals or high-throughput environments where computational resources are constrained. The observed stability across datasets suggests that limited site-specific fine-tuning would be sufficient to achieve consistent performance, reducing barriers to multi-center implementation.

Several considerations nevertheless remain before full clinical translation. Validation on genuinely multi-institutional and multi-vendor MRI cohorts, encompassing broader protocol heterogeneity, is required to confirm robustness under routine clinical operating conditions. Extending the framework toward tumor grading and subtype differentiation would improve clinical relevance, while explicit probability calibration would support safer decision support by aligning confidence estimates with true diagnostic risk. In addition, systematic fairness assessment across demographic and scanner-related factors is necessary to ensure equitable performance.

## 6. Limitations and Future Scope

Despite its strong results, this study has several limitations that merit consideration. First, all three datasets are publicly curated and relatively homogeneous, containing high-quality images with limited acquisition variability. Such controlled conditions may slightly overestimate real-world performance. Future validation should involve multi-center, multi-vendor datasets encompassing diverse MRI protocols, scanner field strengths, and artifact conditions.

Second, the model currently classifies tumors into broad categories, potentially obscuring performance variability across histological subtypes or grades. In clinical contexts, finer-grained classification is often crucial for treatment planning and prognosis.

Third, the exceptionally high accuracy achieved across datasets raises the need for careful data leakage checks at the patient and slice level to ensure that performance gains are not partially influenced by inadvertent overlap.

Fourth, while accuracy and AUC metrics are reported, probability calibration was not explicitly analyzed. Calibration ensures that the model’s confidence estimates correspond to true predictive reliability—an important factor for clinical decision support.

Finally, though interpretability was addressed through Grad-CAM++ and Gradient SHAP visualizations, integrating such methods into the model’s workflow for real-time interpretability remains an open challenge.

Future research will extend this work in several directions. A primary focus will be on external and prospective validation using large, heterogeneous datasets collected from multiple institutions and imaging vendors to confirm the model’s robustness and generalization. The model can also be expanded toward granular tumor classification, incorporating subtype or grade prediction and confidence-based stratification for ambiguous cases. Integrating probability calibration techniques and designing an “uncertain” or abstention mechanism could further reduce false negatives without significantly increasing clinical workload.

Future extensions will explore volumetric and multimodal MRI integration to further enhance spatial reasoning, tumor grading, and progression analysis, particularly in multi-institutional settings where consistent 3D annotations are available.

While the proposed framework demonstrates stable performance across datasets and training runs, the present study does not include an exhaustive sensitivity analysis over alternative preprocessing pipelines. The adopted normalization and augmentation strategy was kept consistent across all experiments to ensure comparability, and indirect evidence of robustness is provided through convergence stability and ablation results. Nevertheless, future work will extend this evaluation to systematically examine the impact of different intensity normalization schemes, scaling strategies, and augmentation policies under broader clinical variability. Such analysis would further characterize robustness across institutions with heterogeneous preprocessing conventions, rather than addressing any instability observed in the current results.

The consistently high accuracy observed across datasets also necessitates consideration of potential data leakage or latent dataset bias. Although training and testing were performed on independent datasets with no shared samples, publicly curated MRI repositories may still contain implicit correlations arising from standardized acquisition protocols, slice-level redundancy within subjects, or prior quality filtering. While cross-dataset evaluation substantially reduces the likelihood of conventional leakage, future work should incorporate explicit patient-level separation checks and validation on large, multi-institutional datasets collected under heterogeneous clinical conditions to further exclude hidden bias.

Beyond categorical tumor classification, the proposed framework provides a flexible foundation for more advanced neuro-oncologic modeling. A direct extension involves integrating tumor segmentation by pairing the ConvNeXt Base encoder with decoder-based architectures, enabling precise localization and volumetric analysis of lesions. Such integration would support surgical planning and longitudinal treatment assessment. In parallel, multi-task learning strategies could be employed to extend the framework toward tumor grading and subtype prediction, allowing shared feature representations to inform multiple clinically relevant outputs. Furthermore, ConvNeXt-derived imaging embeddings could be fused with clinical or genomic data to support radiogenomic prediction, facilitating non-invasive inference of molecular characteristics that currently require biopsy. These extensions build upon the diagnostic reliability and generalization demonstrated in this study and represent natural next steps toward comprehensive, end-to-end clinical decision support systems.

Additional efforts will target explainability integration—using saliency and attention-based interpretive layers (Grad-CAM, LayerCAM, or attention visualizers)—to enable transparent decision-making and reduce automation bias. Stress testing under varied noise, motion, and adversarial perturbations, alongside fairness assessments across demographics (age, sex, scanner type), will also be prioritized to ensure equitable performance.

Finally, practical implementation studies could explore workflow integration, where ConvNeXt Base serves as a triage or quality-assurance tool to flag unreported lesions, prioritize high-risk cases, or support pre-processing for segmentation and molecular marker prediction tasks.

Addressing these aspects represents a logical extension of the present work rather than a correction of its findings, and forms the next step toward clinically integrated deployment.

## 7. Conclusions

This study demonstrates that ConvNeXt Base constitutes a strong and reliable architecture for multi-class brain tumor classification from MRI, achieving consistently high performance across three independent datasets with differing characteristics. Across Figshare (D2), Kaggle (D3), and Other (D4), the model delivered near-perfect or perfect classification across all four clinically relevant categories—Glioma, Meningioma, Pituitary, and No Tumor. The observed accuracies (99.69–99.86%), near-unity AUC values, and high Kappa coefficients confirm that the model is not only accurate but also statistically reliable and class-balanced. Competing architectures such as ResNet152, EfficientNetV2-B0, and VGG19, while competitive, exhibited greater variability, particularly for tumor classes with overlapping radiological appearance.

Class-wise analyses reinforced these findings. The confusion matrices showed consistent diagonal dominance across datasets, indicating stable discrimination between tumor types and a complete absence of systematic confusion between tumor and non-tumor cases. Training and validation trends revealed rapid convergence with negligible divergence, reflecting efficient learning dynamics and strong generalization rather than dataset-specific fitting. ROC analyses further confirmed a robust diagnostic operating profile, demonstrating that ConvNeXt Base sustains high sensitivity and specificity across threshold variations—an essential requirement for clinical screening and decision support.

Equally important, ConvNeXt Base achieved this diagnostic performance without imposing excessive computational cost. Its high inference throughput, moderate memory footprint, and controlled power consumption support deployment on standard clinical GPU infrastructure and, potentially, resource-constrained environments. Ablation experiments showed that performance stability is rooted in the architecture itself: aside from transfer learning—which remains essential for medical imaging—the model maintained strong performance across variations in attention mechanisms, augmentations, and input resolution. This resilience is particularly relevant for multi-center adoption, where training conditions and data characteristics inevitably vary.

Statistical validation provided confirmatory evidence for these empirical observations. Non-parametric tests confirmed that performance differences among architectures are significant and consistent, while ranking agreement across datasets and metrics showed that ConvNeXt Base maintains its position across diverse evaluation perspectives. The convergence of statistical testing, ranking consistency, and multi-criteria analysis indicates that the observed advantages reflect a genuine architectural strength rather than metric-dependent fluctuation.

From a clinical perspective, ConvNeXt Base addresses several persistent barriers to translational adoption: diagnostic reliability, computational feasibility, and interpretability. The integration of Grad-CAM++ and Gradient SHAP confirms that predictions are grounded in anatomically and radiologically meaningful regions, supporting clinician trust and auditability. These properties position ConvNeXt Base as a practical candidate for use in automated triage, second-reader systems, and preprocessing pipelines for downstream neuroimaging tasks.

In its current form, ConvNeXt Base establishes a robust, efficient, and interpretable benchmark for MRI-based brain tumor classification. The consistency of its performance across datasets, metrics, and statistical evaluations positions it as a dependable reference architecture for both comparative research and translational neuroimaging applications.

## Figures and Tables

**Table 1:** Dataset details.

Data Set	Source	Used for	Total Image	Glioma	Meningioma	Pituitary Tumor	No Tumor
D1	https://www.kaggle.com/datasets/masoudnickparvar/brain-tumor-mri-dataset?select=Training, accesses on 15 September 2025	Training & validation	5712	1321	1339	1457	1595
D2	https://figshare.com/articles/dataset/brain_tumor_dataset/1512427, accesses on 15 September 2025	Testing	591	143	152	144	152
D3	https://www.kaggle.com/datasets/masoudnickparvar/brain-tumor-mri-dataset?select=Testing, accesses on 15 September 2025	Testing	1331	300	306	300	405
D4	https://www.kaggle.com/datasets/deeppythonist/brain-tumor-mri-dataset/data?select=test, accesses on 15 September 2025	Testing	1430	324	355	351	400

**Table 2:** Normalization parameters.

Parameter	H, W, C	Description	Purpose
mean	[0.456, 0.406, 0.485]	Per-channel mean pixel values for Red, Green, Blue	Used to align MRI intensity distributions with the normalization statistics of ImageNet-pretrained ConvNeXt weights, ensuring consistent feature scaling during transfer learning and preventing bias from global intensity offsets.
std (standard deviation)	[0.224, 0.225, 0.229]	Per-channel standard deviation for Red, Green, Blue	Applied to normalize per-channel variance, stabilizing gradient flow during training and enabling effective reuse of pretrained convolutional filters under heterogeneous MRI intensity profiles.

**Table 3:** Performance evaluation metrics.

Metrics	Calculation	Clinical Importance
Accuracy	$\frac{TP+TN}{FP+FN+TP+TN}$	Reflects the overall proportion of correctly classified MRI scans across all tumor and non-tumor categories. In brain tumor diagnosis, high accuracy suggests that the model reliably distinguishes between normal and abnormal cases. However, accuracy alone can be misleading when one class (e.g., “No Tumor”) dominates, since a model may appear accurate while missing rare but critical tumor cases.
Precision	$\frac{TP}{TP+FP}$	Indicates how many MRI scans identified as tumors actually contain a lesion. In neuro-oncology, high precision means fewer false alarms—reducing unnecessary follow-up MRIs, invasive procedures, and patient anxiety caused by incorrect tumor alerts. It ensures the model’s positive predictions correspond to clinically meaningful findings.
Recall	$\frac{TP}{TP+FN}$	Represents the ability to correctly detect true tumor cases among all tumors present. High recall is critical for brain tumor diagnosis because false negatives correspond to missed lesions, which may delay treatment or surgery. A sensitivity close to 1.0 ensures that even small or atypical tumors are not overlooked during automated screening.
F1-score	$2\times \frac{\mathrm{Precision}\times \mathrm{Recall}}{\mathrm{Precision}+\mathrm{Recall}}$	Provides a harmonic balance between precision and recall. In clinical use, a high F1-score confirms that the system detects most tumors while maintaining low false-positive rates. This balance minimizes both missed diagnoses and unnecessary alerts, supporting dependable triage and reporting in radiology workflows.
Area under the curve (AUC)	$\int_{0}^{1}TPR\left(FPR\right)d(FPR)$	Reflects the model’s overall discriminative capacity between tumor and non-tumor scans. In clinical MRI classification, an AUC near 1.0 means the algorithm can reliably separate abnormal brains from healthy ones across diagnostic thresholds. This implies the model’s decision threshold can be safely adjusted depending on whether the goal is broad screening (favoring sensitivity) or confirmatory diagnosis (favoring specificity).
Support	Count of instances per class used to compute metrics	Represents how many MRI scans belong to each tumor class (Glioma, Meningioma, Pituitary, No Tumor). In medical evaluation, this provides context for interpreting metric stability—metrics based on smaller tumor categories should be interpreted cautiously due to higher statistical variance.
Cohen’s Kappa (κ)	$\frac{p_{o}-p_{e}}{1-p_{e}}$ $,\;\mathrm{where}\;p_{o}=\frac{TP+TN}{N}$ $\;\mathrm{and}\;p_{e}=\frac{\left(TP+FP)(TP+FN)+(FN+TN)(FP+TN\right)}{N^{2}}$	Measures the agreement between the model’s predictions and the radiologically verified ground truth beyond chance. A κ value approaching 1.0 indicates near-perfect diagnostic consistency, equivalent to agreement between two expert radiologists. In brain tumor classification, high κ supports the model’s reproducibility and its potential for routine clinical decision support.

TP: True positives, FP: False positives, FN: False negatives, TN: True negatives, TPR: TP/(TP + FN), FPR: FP/(FP + TN), N: Total number of samples.

**Table 6:** Class-wise and average classification performance of ConvNeXt Base and baseline models across D2, D3, and D4.

Data Set	Model	Precision				Recall				F1-Score				Support			
		Glioma	Meningioma	No_Tumor	Pituitary	Glioma	Meningioma	No_Tumor	Pituitary	Glioma	Meningioma	No_Tumor	Pituitary	Glioma	Meningioma	No_Tumor	Pituitary
D2	MobileNetV3Large	99.31	96.92	100	99.68	96.51	99.02	100	100	97.85	97.91	100	99.84	143	152	144	152
	ResNet152	99.30	96.75	99.65	98.72	98.60	98.03	98.27	99.34	98.95	97.38	98.95	99.02	143	152	144	152
	EfficientNetV2-B0	99.65	98.39	100	99.03	98.25	99.67	98.96	100	98.94	99.02	99.48	99.51	143	152	144	152
	VGG19	98.91	95.95	100	97.15	95.81	98.69	96.88	100	99.30	54.34	99.30	99.35	143	152	144	152
	ConvNeXt Base	100	99.68	100	99.68	99.65	99.67	100	100	99.83	99.67	100	99.84	143	152	144	152
D3	MobileNetV3Large	99.16	95.87	99.27	99.00	97.50	97.55	99.38	98.67	98.31	96.67	99.32	98.83	300	306	405	300
	ResNet152	97.72	94.99	99.75	99.47	99.00	98.37	99.63	94.67	98.35	96.64	99.69	97.01	300	306	405	300
	EfficientNetV2-B0	98.84	97.88	99.75	98.04	98.84	96.90	99.63	99.17	98.84	97.37	99.69	98.60	300	306	405	300
	VGG19	98.04	96.05	99.76	98.49	99.67	96.57	99.26	96.84	98.85	96.27	99.51	97.63	300	306	405	300
	ConvNeXt Base	99.67	99.35	100	99.67	99.84	99.35	99.75	99.84	99.75	99.35	99.88	99.75	300	306	405	300
D4	MobileNetV3Large	99.39	99.15	99.50	99.86	99.38	99.02	99.63	99.86	99.39	99.08	99.56	99.86	324	355	400	351
	ResNet152	97.27	96.97	98.48	98.22	98.30	95.63	97.75	99.43	97.78	96.29	98.12	98.81	324	355	400	351
	EfficientNetV2-B0	98.20	99.30	99.50	99.86	99.54	99.39	99.75	99.72	98.86	98.58	99.63	99.79	324	355	400	351
	VGG19	99.38	98.48	99.88	99.01	98.46	98.73	99.50	100	99.41	98.60	99.69	99.50	324	355	400	351
	ConvNeXt Base	100	99.72	99.75	100	99.69	100	99.75	100	99.85	99.86	99.75	100	324	355	400	351

**Table 7:** Average performance of four classes for five models on three datasets.

Dataset	Model	Accuracy	Precision	Recall	F1-Score	AUC	Kappa
D2	MobileNetV3Large	98.90	98.98	98.88	98.90	99.97	98.48
	ResNet152	98.56	98.60	98.56	98.57	99.81	97.37
	EfficientNetV2-B0	99.24	99.27	99.22	99.24	100	98.87
	VGG19	97.89	98.00	97.84	88.07	99.83	95.26
	ConvNeXt Base	**99.83**	**99.84**	**99.83**	**99.83**	**100**	**99.77**
D3	MobileNetV3Large	98.36	98.32	98.27	98.28	99.91	98.08
	ResNet152	98.06	97.98	97.92	97.92	99.68	95.60
	EfficientNetV2-B0	98.70	98.63	98.63	98.62	99.97	97.95
	VGG19	98.17	98.08	98.08	98.06	99.80	96.93
	ConvNeXt Base	**99.69**	**99.67**	**99.69**	**99.68**	**99.98**	**99.59**
D4	MobileNetV3Large	99.76	99.47	99.47	99.47	100	99.72
	ResNet152	97.76	97.73	97.78	97.75	99.62	94.77
	EfficientNetV2-B0	98.73	99.22	99.60	99.21	99.99	98.69
	VGG19	99.20	99.19	99.17	99.30	99.96	98.60
	ConvNeXt Base	**99.86**	**99.87**	**99.86**	**99.87**	**100**	**99.86**

**Table 8:** Computational efficiency comparison of ConvNeXt Base and baseline models averaged across three datasets.

Model	Inference Speed (FPS)	Model Size (MB)	GPU Memory Allocated (MB)	GPU Memory Reserved (MB)	CPU Memory Usage (MB)	Power Consumption (Watt)
MobileNetV3Large	352.62	16.25	1505.40	16,060.00	3724.34	52.02
ResNet152	358.71	222.71	2956.48	16,292.00	3729.89	59.12
EfficientNetV2-B0	353.94	22.75	1897.77	16,292.00	3755.02	55.59
VGG19	362.73	532.49	4476.24	19,840.00	3760.45	72.31
ConvNeXt Base	370.88	334.18	1703.67	9684.00	2635.61	59.13

**Table 11:** Friedman aligned ranks test statistics for three test sets.

Dataset	Statistic	*p*-Value	Result
D2	18.93142	0.00081	H0 rejected
D3	19.97818	0.00050	H0 rejected
D4	18.46859	0.00100	H0 rejected

**Table 12:** Model-wise ranking of Friedman aligned test across three test sets.

Rank	D2		D3		D4	
	Rank Score	Algorithm	Rank Score	Algorithm	Rank Score	Algorithm
1	4.33333	VGG19	5.66667	ResNet152	3.66667	ResNet152
2	11.00000	ResNet152	8.66667	VGG19	13.66667	VGG19
3	17.16667	MobileNetV3Large	15.33333	MobileNetV3Large	14.66667	EfficientNetV2-B0
4	20.91667	EfficientNetV2-B0	20.75000	EfficientNetV2-B0	21.08333	MobileNetV3Large
5	24.08333	ConvNeXt Base	27.08333	ConvNeXt Base	24.41667	ConvNeXt Base

**Table 13:** Holm’s post hoc pairwise comparison of the considered models across datasets.

Comparison	Statistic			Adjusted *p*-Value			Result		
	D2	D3	D4	D2	D3	D4	D2	D3	D4
ConvNeXTBase vs. VGG19	3.88577	3.62344	2.11504	0.00102	0.00262	0.24358	H0 rejected	H0 rejected	H0 accepted
EfficientNetV2-B0 vs. VGG19	3.26273	2.37737	0.19675	0.00993	0.12206	1.00000	H0 rejected	H0 accepted	H0 accepted
ResNet152 vs. ConvNeXTBase	2.57412	4.21368	4.08252	0.08040	0.00025	0.00045	H0 accepted	H0 rejected	H0 rejected
MobileNetV3Large vs. VGG19	2.52493	1.31165	1.45921	0.08101	0.75855	0.57803	H0 accepted	H0 accepted	H0 accepted
EfficientNetV2-B0 vs. ResNet152	1.95108	2.96761	2.16423	0.30628	0.02401	0.24358	H0 accepted	H0 rejected	H0 accepted
MobileNetV3Large vs. ConvNeXTBase	1.36084	2.31179	0.65583	0.86782	0.12474	1.00000	H0 accepted	H0 accepted	H0 accepted
ResNet152 vs. VGG19	1.31165	0.59024	1.96748	0.86782	0.75855	0.29477	H0 accepted	H0 accepted	H0 accepted
MobileNetV3Large vs. ResNet152	1.21328	1.90189	3.42669	0.86782	0.28592	0.00550	H0 accepted	H0 accepted	H0 rejected
EfficientNetV2-B0 vs. MobileNetV3Large	0.73780	1.06572	1.26246	0.92127	0.75855	0.62034	H0 accepted	H0 accepted	H0 accepted
EfficientNetV2-B0 vs. ConvNeXTBase	0.62303	1.24607	1.91829	0.92127	0.75855	0.29477	H0 accepted	H0 accepted	H0 accepted

**Table 14:** Pairwise Wilcoxon signed-rank test results for ConvNeXt Base versus other models across datasets.

Comparison	Statistic			Adjusted *p*-Value			Result		
	D2	D3	D4	D2	D3	D4	D2	D3	D4
ConvNeXt Base vs. MobileNetV3Large	0	0	0	0.02728	0.02771	0.04217	H0 rejected	H0 rejected	H0 rejected
ConvNeXt Base vs. ResNet152	0	0	0	0.02728	0.02771	0.02771	H0 rejected	H0 rejected	H0 rejected
ConvNeXt Base vs. EfficientNetV2-B0	0	0	0	0.04217	0.02728	0.02771	H0 rejected	H0 rejected	H0 rejected
ConvNeXt Base vs. VGG19	0	0	0	0.02771	0.02771	0.02771	H0 rejected	H0 rejected	H0 rejected

**Table 15:** TOPSIS-based multi-criteria ranking of models across accuracy, reliability, and efficiency metrics.

Model	D2		D3		D4	
	TOPSIS Score	TOPSIS Rank	TOPSIS Score	TOPSIS Rank	TOPSIS Score	TOPSIS Rank
Convnext-Base	1	1	1	1	1	1
Efficient-NetV2B0	0.890715	2	0.50831	2	0.722415	4
MobileNetV3Large	0.83749	3	0.45277	3	0.89677	2
ResNet152	0.757662	4	0	5	0	5
VGG19	0.001492	5	0.24811	4	0.727245	3

**Table 16:** Comparison of ConvNeXt Base with recent state-of-the-art methods for brain tumor classification on MRI.

Reference	Dataset Used	Image Size	No. of Classes	Considered Models	Model with Best Accuracy	Accuracy (%)	Precision (%)	Recall (%)	F1-Score (%)	AUC	Kappa	XAI	Statistical Analysis	Ablation Study	Efficiency Comparison	MCDM Ranking
Reyes & Sánchez [16]	Figshare dataset	3064	4	VGG16, VGG19, ResNet50, EfficientNet, DenseNet, Xception	EfficientNet	95.7	-	-	-	-	-	X	X	X	√	X
	Kaggle dataset	3264	4			98.8	-	-	-	-	-					
Pravitasari et al. [22]	16 test datasets	-	4	UNet and VGG16	UNet-VGG16	96.1	-	-	-	-	-	X	X	X	X	X
Nayak et al. [23]	Kaggle dataset	3064	4	EfficientNet, ResNet50, MobileNet, MobileNetV2	EfficientNet	98.78	98.75	98.75	99.00	-	-	X	X	X	X	X
Salama & Shokry [27]	Chakrabarty N (2019) dataset for brain tumor MRI images from Kaggle	253 samples	2	CNN, transfer learning, proposed model	Proposed model	96.88	96.88	96.88	96.88	-	-	X	X	X	X	X
Sharif et al. [29]	BRATS2018 dataset	335 cases	4	Densenet201	-	99.7	99.7	99.7	99.7	0.99	-	X	X	X	X	X
	BRATS2019 dataset		4			99.8	99.8	99.8	99.8	1.00	-	X	X	X	X	X
Bhatti et al. [48]	BraTS2019 dataset	22,150	4	ConVNeXT	-	99.5	-	-	-	-	-	X	X	X	X	X
Nahiduzzaman et al. [52]	Kaggle dataset	7023	4	PDSCNN-RELM, PELM	PELM	99.21	99.0	99.50	0.99	0.99	-	SHAP	X	X	X	X
Moodely et al. [57]	Kaggle dataset	7023	4	SeparableConvNet, ConvNet,	SeparableConvNet	96.64	-	-	-	-	-	LIME	X	X	X	X
Singh et al. [58]	Kaggle dataset	22,000	4	ResNet-50, EfficientNet-B5, Custom CNN	ResNet-50, EfficientNet-B5 and Custom CNN	99.0	99.25	98.75	99.5	-	-	Grad-CAM, LIME, SHAP, SmoothGrad	X	X	X	X
Ferdous et al. [66]	Figshare dataset	233	3	ResNet-50, ViT, Swin, DEiT, LCDEiT	LCDEiT	98.11	97.86	97.84	97.85	0.99	-	X	X	√	X	X
	BraTS2021 dataset	2040	4			93.69	93.68	93.68	93.68	0.97	-					
Almuhaimeed et al. [67]	Kaggle dataset	3000	4	AutoEncoder + Swin	-	99.54	99.54	99.54	99.54	0.99	-	X	X	X	X	X
	Figshare dataset	3064	4			98.90	98.90	98.90	98.90	0.99	-					
Alam et al. [68]	Kaggle dataset	7023	4	Swin V2, ResNet50, DenseNet121	Improved Swin V2	98.97	98.75	98.51	98.63	-	-	X	X	X	X	X
Srinivas et al. [69]	Kaggle dataset	7023	4	DTLA, AGAN, MLGAN, SbCNN, DCNN	DeiT, Firefly algorithm	99.70	99.60	99.80	99.50	-	-	X	X	X	X	X
Shanto et al. [70]	Kaggle dataset	7023	4	ResNet50, Transformer encoder	Hybrid CNN-Transformer	99.00	100	100	99.00	-	-	Grad-CAM	X	X	X	X
Pacal [71]	Kaggle dataset	7023	4	MobileViTv2-150, BeiT-Base, MobileViT-Small, DeiT-Base, with ResNet50 and other Swin variants	Swin	99.92	99.92	99.92	99.92	-	-	X	X	X	√	X
Iftikhar et al. [72]	Kaggle dataset	7023	4	XAI-based CNN model	-	99.21	99.22	99.18	99.20	-	-	Grad-CAM, SHAP, LIME	X	X	X	X
Mala et al. [73]	BraTS2020 dataset	369	4	U-Net	Multi-scale attention U-Net	99.39	99.42	99.26	99.34	-	-	Grad-CAM, Saliency maps, Vanilla Grad, MRP	X	X	√	X
Bataineh et al. [74]	BraTS2021 dataset	3160	4	ResNET50V2, VGG16, MobileNetV2, CNN, ConVNeXTTiny, EfficientNetV2B3	SwT+Resnet50V2	96.80	-	97.00	97.00	-	-	X	√	X	X	X
	BrH35 dataset	3000	4			99.90	-	99.90	99.90	-	-					
Farhan et al. [75]	BraTS2020 dataset	494	3	U-Net, U-Net++, Attention U-Net	Ensemble dual-modality U-Net	98.36	98.80	98.03	97.73	-	-	Grad-CAM	X	X	X	X
Hasan et al. [76]	BrainTumorInSight	1625	2	ResNet50, InceptionV3, VGG16, DenseNet121, EfficientNet	ResNet50	98.03	85.02	86.74	85.80	-	-	LIME	X	X	X	X
Zeineldin [77]	BraTS2019 dataset	335	2	ResNet50	-	98.62	-	-	-	-	-	SmoothGrad, Grad-CAM, guided Grad-CAM	X	X	X	X
Nazir et al. [78]	BR35H dataset	3060	2	Customized CNN	-	98.67	98.50	98.50	98.50	0.98	-	SHAP, LIME	X	X	X	X
	NeuroMRI dataset	3264	4			94.72	94.70	95.10	94.63	-	-					
Lakshmi et al. [79]	BT-MRI dataset from Kaggle	5500	4	UNet	XAISS-BMLBT	97.75	95.56	95.42	95.48	0.98	-	X	X	X	X	X
Kumar et al. [80]	Collection of ACRIN-DSC-MR-Brain (ACRIN 6677/RTOG 0625), CPTAC-GBM, and ACRIN-FMISO-Brain (ACRIN 6684)	1572	2	Res-Net, Alex-Net, U-Net, VGG-16	Novel CNN ResNet50	98.85	97.50	96.38	97.34	-	-	X	X	X	X	X
Asiri et al. [81]	Figshare dataset	2870	4	Swin	-	97.00	96.00	96.25	96.00	-	-	X	X	X	X	X
This paper	Figshare dataset	591	4	MobileNetV3Large, ResNet50, VGG19, EfficientNetV2-B0, ConvNeXt Base	ConvNeXt Base	99.83	99.84	99.83	99.83	1.00	0.99	Grad CAM++, Gradient SHAP	√	√	√	TOPSIS
	Kaggle dataset	1331	4			99.69	99.67	99.69	99.68	0.99	0.99					
	Kaggle dataset	1430	4			99.86	99.87	99.86	99.87	1.00	0.99					

## Data Availability

The datasets used in the experiment are publicly available at https://github.com/Kirti-Pant/ConvNeXT-Base-for-Brain-Tumor-Classification--Data.git. The code used in this study is openly available on GitHub at https://github.com/Kirti-Pant/ConvNeXT-Base-for-Brain-Tumor-Classification.git.
